# Extracellular Vesicles’ Genetic Cargo as Noninvasive Biomarkers in Cancer: A Pilot Study Using ExoGAG Technology

**DOI:** 10.3390/biomedicines11020404

**Published:** 2023-01-30

**Authors:** Carolina Herrero, Alba Ferreirós, Daniel Pérez-Fentes, Luis León-Mateos, Rafael López-López, Miguel Abal, Lorena Alonso-Alconada

**Affiliations:** 1 Translational Medical Oncology Group (Oncomet), Health Research Institute of Santiago de Compostela (IDIS), University Hospital of Santiago de Compostela (SERGAS), CIBERONC, 15706 Santiago de Compostela, Spain; 2 Nasasbiotech, S.L., Canton Grande 9, 15003 A Coruña, Spain; 3 Urology Department, University Hospital of Santiago de Compostela (SERGAS), 15706 Santiago de Compostela, Spain

**Keywords:** extracellular vesicles, ExoGAG, EV-DNA, EV-mRNA, prostate cancer

## Abstract

The two most developed biomarkers in liquid biopsy (LB)—circulating tumor cells and circulating tumor DNA—have been joined by the analysis of extracellular vesicles (EVs). EVs are lipid-bilayer enclosed structures released by all cell types containing a variety of molecules, including DNA, mRNA and miRNA. However, fast, efficient and a high degree of purity isolation technologies are necessary for their clinical routine implementation. In this work, the use of ExoGAG, a new easy-to-use EV isolation technology, was validated for the isolation of EVs from plasma and urine samples. After demonstrating its efficiency, an analysis of the genetic material contained in the EVs was carried out. Firstly, the sensitivity of the detection of point mutations in DNA from plasma EVs isolated by ExoGAG was analyzed. Then, a pilot study of mRNA expression using the nCounter NanoString platform in EV-mRNA from a healthy donor, a benign prostate hyperplasia patient and metastatic prostate cancer patient plasma and urine samples was performed, identifying the prostate cancer pathway as one of the main ones. This work provides evidence for the value of using ExoGAG for the isolation of EVs from plasma and urine samples, enabling downstream applications of the analysis of their genetic cargo.

## 1. Introduction

Precision medicine based on liquid biopsy (LB) contributes to early diagnosis, detection of recurrence and minimal residual disease [1] allowing for real-time analysis of the mutational profile of the tumor, which provides crucial information for the selection of the most effective therapies. It has been shown that patients treated with therapy selected on the basis of molecular alterations present in LB samples have a significant increase in progression-free survival compared to patients who did not receive matched therapy [2]. In addition, liquid biopsy can be used to identify markers for stratifying patients [3] and provides a solution to the problem of intratumoral heterogeneity and tumor evolution that contribute to treatment failure. Furthermore, in the case of patients with metastatic disease, where metastases have resisted different treatments, LB offers the advantage of avoiding successive re-biopsies.

This strategy includes the study of circulating tumor cells (CTC), the so-called cell-free nucleic acids in plasma and serum (CNAPS), extracellular vesicles (EVs), tumor-educated platelets, proteins and metabolites present in biofluids [4], providing a powerful clinical tool for diagnosis, for the selection of specific molecularly targeted therapies and for the monitoring of the disease. 

The analysis of CTC, a rare subset of cells found in the blood of patients with solid tumors, could help to better elucidate the mechanisms favoring tumor spread and dissemination [5], and their molecular characterization allows us to obtain information for targeted therapy selection in the treatment of metastatic cancer patients [6,7,8]. Additionally, the assessment of an RNA signature in CTC may be useful in the identification of noninvasive potential biomarkers of early response or resistance to therapy [9,10]. Nevertheless, despite the advances in the field of CTC, to date, the CellSearch^®^ system is the only FDA-approved method for the enumeration of EpCAM-expressing CTC in which the presence of CTC is associated with decreased progression-free survival (PFS) and overall survival (OS) in patients treated for metastatic breast, colorectal or prostate cancer [11]. On the other hand, the term “CNAPS” refers to different types of cell-free nucleic acids, such as genomic DNA (gDNA), cell-free DNA (cfDNA), circulating tumor DNA (ctDNA), mitochondrial DNA (mitDNA), messenger RNA (mRNA) and microRNA (miRNA), reported to be present in plasma [12]. Changes in the levels of cfDNA, ctDNA, mRNA and microRNA, released by passive mechanisms, including apoptosis and necrosis, or active mechanisms into the blood of cancer patients have been associated with tumor burden and malignant progression. In general, although the majority of cfDNA (extracellular double-stranded DNA and mitDNA) is often of non-cancerous origin, its levels are frequently elevated in the blood of cancer patients and its kinetics may be useful to assist in cancer diagnosis, treatment response or prognostic prediction [13,14,15,16]. ctDNA has the potential to be used in early cancer detection, and the levels of ctDNA may also have a prognostic value even after surgery, detecting minimal residual disease [17,18]. However, one of its greatest strengths lies in the detection of specific mutations that provide information about the selection of therapies, to monitor response and resistance to treatment. As in the case of CTC, with relatively low amounts of mutated ctDNA in a background of normal cfDNA, a very sensitive and specific approach is required, posing a significant challenge in the development of liquid biopsy applications. 

Another pillar of liquid biopsy is extracellular vesicles (EVs), a heterogeneous group of membranous structures released by all cell types and classified into 3 major subtypes: exosomes (30–100 nm), microvesicles (100–1000 nm) and apoptotic bodies (1–5 μm). This type of nanostructure carries the molecular signature of the parental cells, including nucleic acids (EV-DNA, EV-mRNA and EV-miRNA, among others), proteins and lipids, which can be transported locally or over distance,, becoming the main mediators of intercellular communication [19]. EVs play an important role in tumor development and progression, especially in the establishment of premetastatic niches [20,21], and their levels have been shown to be elevated in cancer, where their secretion correlates with tumor invasiveness [22]. In addition, an important feature of EVs is the presence of a double membrane, which confers stability to the genetic material contained within the EVs, protecting it from nucleases present in plasma and other biological fluids. In this regard, EVs have emerged as potential prognostic and diagnostic biomarkers to improve the management of different type of tumors. In NSCLC patients, the detection of *EGFR T790M* mutation on exosomal DNA (exoDNA) from plasma samples has shown better sensitivity and specificity compared to cfDNA for early diagnosis [23,24]. In pancreatic cancer, detection of *KRAS* and *TP53* mutations in exoDNA has been shown to distinguish cancer patients and healthy controls [25], an added advantage to the fact that new therapies targeting p53 mutations are being described [26]. This data demonstrated that EV-DNA could be a promising diagnostic tool based on its increased sensitivity, specificity, and stability to carry information about cancer-specific mutations.

Circulating RNA in biological fluids has also emerged as a new field in which to explore gene expression noninvasively. RNAs are not stable in biofluids and usually are associated with proteins such as Ago2 [27] with HDL and LDL particles [28], or protected from RNases in the enclosed lipid bilayer structure of EVs [29]. In fact, EV-RNAs have emerged as a potential diagnostic and prognostic tool for cancer management and therapy efficacy monitoring due to their stability in biological fluids [30,31]. In this regard, a new technology based on EV-RNA has recently been developed to improve the diagnosis of prostate cancer patients. ExoDx^®^ Prostate (IntelliScore) is a urine exosome gene expression test approved by the US Food and Drug Administration (FDA) to discriminate high-grade from low-grade cancer and benign disease. This strategy combines the analysis of the expression levels of three RNAs (PCA3 ncRNA, ERG mRNA and SPDEF mRNA) directly detected in urinary EVs [32].

EVs have certain advantages over other types of liquid biopsy since, in addition to being present in all biological fluids, they possess great stability due to their lipid bilayer protection. However, despite their potential, one of the limitations of EV-based biomarkers is the absence of an efficient isolation and enrichment method that can be easily implemented into clinical routine; currently, the most widespread method is differential ultracentrifugation. To overcome this barrier, we developed ExoGAG, an extracellular vesicle purification kit that can be directly implemented in the clinic. In previous studies, we have demonstrated that ExoGAG is a robust and highly efficient isolation technology with optimal performance for clinical application of EVs, mainly through the protein analysis of EV content [33,34]. 

Now, in this work, we have evaluated the potential of the EVs isolated with ExoGAG for the subsequent analysis of their nucleic acids content (EV-DNA, EV-mRNA and EV-miRNA) as a technology for EV-based biomarker discovery in liquid biopsy samples. For this purpose, we have first isolated EVs by ExoGAG from plasma and urine samples. Next, we have evaluated the efficiency and sensitivity in the detection of point mutations in the DNA contained in EVs isolated by ExoGAG. Moreover, we have optimized an EV-mRNA purification protocol and performed a proof-of-concept assay to analyze the EV-mRNA expression of 770 genes represented on NanoString’s PanCancer gene expression panel. Finally, we have applied ExoGAG technology in the detection of miRNAs contained in EVs from plasma samples. Therefore, in this pilot study we have demonstrated that ExoGAG allows the isolation of EVs from plasma and urine samples suitable for downstream analysis of their nucleic acids content, with potential application in liquid biopsy-based precision medicine.

## 2. Materials and Methods

### 2.1. Isolation of EVs from Plasma and Urine Samples Using ExoGAG

This study was approved by the Autonomic Galician Ethical Committee (code 2017/430). Peripheral blood was collected in EDTA Vacutainer tubes (BD Vacutainer, 367525, Plymouth, UK) and centrifuged for 10 min at 1600× *g.* The supernatant was collected and centrifuged again for 10 min at 5500× *g* to remove remaining cells and stored at −80 °C until use. Urine samples were collected in C&S Boric Acid Sodium Borate/Formate tubes (BD Vacutainer, 364955, Eysins, Switzerland), centrifuged for 10 min at 3000× *g* to remove any cellular debris and stored at −80 °C until use. EVs were isolated from plasma and urine samples using ExoGAG technology (Nasasbiotech, A Coruña, Spain), as previously described [33]. Plasma samples were incubated with twice the volume of ExoGAG (1:2; plasma:ExoGAG), while urine samples with half the volume of reagent (1:0,5; urine:ExoGAG) for 5 min at 4 °C. Then, EVs were collected by a centrifugation step at 3000× *g* for 15 min at 4 °C and the pellet was re-suspended in PBS (D8537, Sigma Aldrich, St. Louis, MO, USA) for EV characterization (NTA Nanosight NS300 and flow cytometry) or in lysis buffer to isolate EV-DNA, EV-mRNA or EV-miRNA (EVs’ nucleic acid analysis).

### 2.2. EVs’ Production and Isolation from HCT116 Human Colorectal Cancer Cell Line by Ultracentrifugation

The human colorectal cancer (CRC) cell line HCT116 (ATCC, CCL-247) that harbors a heterozygous *PIK3CA H1047R* mutation was cultured in McCoy’s 5A media (Gibco, Grand Island, NY, USA) supplemented with 1% penicillin-streptomycin (Gibco, Grand Island, NY, USA) and 10% FBS (Gibco, Thermo Fisher Scientific) reduced in EVs by ultracentrifugation during 16 h at 4 °C, as previously described [35]. HCT116 cell line was maintained in a humidified atmosphere at 37 °C and 5% CO_2_. After 48 h, 38 mL of the conditioned medium was recovered and EVs were harvested by differential centrifugation with the following conditions: 500× *g*, 10 min; 10,000× *g*, 20 min, 4 °C followed by an ultracentrifugation at 100,000× *g*, 16 h, 4 °C using a SW32Ti rotor (Beckman Coulter, Brea, CA, USA). EVs were re-suspended in PBS and stored at −80 °C until use.

### 2.3. Characterization of EVs Isolated from Plasma and Urine Samples Using ExoGAG Technology

Plasma and urine EVs purified by ExoGAG were characterized by NTA Nanosight NS300 and flow cytometry assays. After isolation, EVs from urine samples required a uromodulin depletion pre-treatment for NTA Nanosight and flow cytometry characterization assays, not for nucleic acids purification. For this, ExoGAG-EV pellet was incubated with dithiothreitol (DTT, final concentration 200 mg/mL) for 10 min at 37 °C and 1000 rpm. Then, a centrifugation at 3000× *g*, 4 °C for 15 min was performed. The supernatant was discarded, and the pellet was re-suspended in PBS for characterization.

#### 2.3.1. NTA Nanosight NS300 (NTA)

NTA Nanosight NS300 (NTA) technology (Marlvern, UK) uses the properties of both light scattering and Brownian motion to obtain measurements of concentration and particle size distribution in a liquid suspension. An amount of 50 μL of plasma and 3 mL of urine samples were used to isolate EVs by ExoGAG, as previously described. The EV pellet was re-suspended in 1mL PBS (D8537, Sigma Aldrich, St. Louis, MO, USA) and analyzed with the NTA using a 405 nm wavelength laser. EVs were measured at 11 camera level for plasma samples and 13 for urine samples. The videos were analyzed using the NanoSight Software NTA 3.4.003 with a detection threshold of 17.

#### 2.3.2. Flow Cytometry Analysis

An amount of 100 μL of plasma and 3 mL of urine samples were used for EV isolation using ExoGAG. The pellet containing EVs was resuspended in 200 μL of PBS and incubated for 1 h at 4 °C with antibodies against EVs’ protein markers [36]: CD9 (1:50, sc13118, Santa Cruz Biotechnology, Santa Cruz, CA, USA), CD63 (1:50, sc5275, Santa Cruz Biotechnology, Santa Cruz, CA, USA) and flotillin-1 (1:50, sc74566, Santa Cruz Biotechnology, Santa Cruz, CA, USA). An isotype IgG antibody (1:50, 400120, BioLegend, San Diego, CA, USA) was used as negative control and added in equivalent volumes. Subsequently, an anti-mouse Alexa 488 (1:1000, ab150113, Abcam, Cambridge, UK) secondary antibody was added and stained for 1h at 4 °C in the dark. EVs were then washed once with PBS by centrifugation at 3000× *g* for 15 min at 4 °C and analyzed by flow cytometry (BD FACSAriaTM IIu, BD biosciences, San José, CA, USA).

### 2.4. EV-DNA Purification and Droplet Digital PCR (ddPCR) Analysis

EVs were isolated using ExoGAG from 3 mL of healthy donors’ plasma samples enriched with previously isolated HCT116-EVs by ultracentrifugation: 1.29 × 10^6^, 6.45 × 10^5^ and 1.29 × 10^5^, to confirm the ExoGAG isolation efficacy and sensitivity by point mutation detection using ddPCR. In addition, EVs were isolated by ExoGAG from 3 mL of plasma samples from CRC patients with a previously known point mutation (*KRAS G12V*). EV-DNA was purified using the QIAamp Circulating Nucleic Acid Kit (CNA) (Qiagen, Hilden, Germany), according to manufacturer’s instructions, and *PIK3CA H1047R* (HCT116-EVs) and *KRAS G12V* (CRC patients) mutations were analyzed by ddPCR, according to the manufacturer’s instructions (Bio-Rad, Hercules, CA, USA): a 12.6 μL reaction mixture of 6 μL ddPCR Supermix for Probes (Bio-Rad, CA, USA), 0.6 μL PrimePCR ddPCR assay (*PIK3CA H1047R* (dHsaMDV2010077, Bio-Rad, CA, USA) or *KRAS G12V* (dHsaMDV2510592, Bio-Rad, CA, USA)), 5.4 μL of EV-DNA sample and 0.6 μL of HaeIII enzyme (Takara Bio, CA, USA). Droplets were generated using a QX200 AutoDG Droplet Digital PCR System (Bio-Rad, Hercules, CA, USA) with Droplet Generation Oil for Probes (Bio-Rad, Hercules, CA, USA) and transferred to a 96-well plate. PCR amplification was performed using the following conditions: 95 °C for 10 min, 40 cycles of 94 °C for 30 s, 55 °C for 60 s and 10 min incubation at 98 °C. The plates were read on a Bio-Rad QX200 droplet reader (Bio-Rad, Hercules, CA, USA) and mutant allele frequency (MAF) was analyzed using the QuantaSoft Pro software (Bio-Rad, Hercules, CA, USA).

### 2.5. EV-RNA Purification and Quantitative Real-Time PCR (RT-q-PCR)

EVs were purified by ExoGAG from 3 mL of plasma and urine samples. Total EV-RNA was extracted using three commercial kits: QIAamp Circulating Nucleic Acid Kit (CNA) (Qiagen, Hilden, Germany) and RNeasy Mini Kit (Qiagen, Hilden, Germany), according to the manufacturer’s instructions, and the DNeasy Blood and Tissue Kit (DBT) (Qiagen, Hilden, Germany), with some protocol modifications (see Figure S1). In parallel, these three kits were compared with the addition of a previous lysis step with TRIzol (TRIzol™ Reagent, 15596018, Thermofisher Scientific, Van Allen Way, CA, USA) and chloroform (J.T Baker, Gliwice, Poland). An amount of 750 μL of TRIzol for every 500 μL of sample were added, mixed thoroughly and incubated at room temperature (RT) for 5 min. Then, 200 μL of chloroform for every 500 μL of sample were added, mixed thoroughly and incubated at RT for 5 min. The aqueous upper phase containing the RNA was collected after centrifugation at 12,000× *g* for 15 min at 4 °C and then each protocol was followed according to the instructions. Treatment with DNase I (79254, Qiagen, Hilden, Germany) was carried out, according to manufacturer’s protocol.

After RNA purification, cDNA was synthesized using MuLV reverse transcriptase (Applied Biosystems, Foster City, CA), following manufacturer’s protocol. RT-q-PCR was performed using a *GAPDH* TaqMan assay (#Hs99999905_m1; Applied Biosystem, Foster City, CA, USA) on a QuantSudio^TM^ 3 instrument (Thermofisher Fisher Scientific, Waltham, MA, USA) and following the conditions: 50 °C for 2 min, 95 °C for 10 min, 40 cycles (95 °C for 15 s and 60 °C for 1 min). The results of *GAPDH* gene expression were represented as 40-Cq Mean. Data were analyzed with the Quantstudio™ Design & Analysis software, version 2.5.1 (Thermofisher Fisher Scientific, Waltham, MA, USA). 

### 2.6. Gene Expression Analysis Using nCounter NanoString

EVs were isolated by ExoGAG from 6 mL of healthy donor (control), benign prostatic hyperplasia (BPH) and metastatic prostate cancer (mPC) plasma samples. EVs from 7 mL of urine from the same mPC patient were also purified by ExoGAG, as previously described. For nucleic acid purification, urine processing did not require DTT treatment. The addition of the reducing agent DTT to the pellet could disrupt the polymeric uromodulin network and improve the access to specific antibodies for EVs’ characterization. However, RNA yield from urinary EVs does not increase after DTT treatment [37]. EV-RNA was purified using DBT Kit with a previous TRIzol-chloroform lysis step. A 10-cycles pre-amplification step was performed after retro-transcription with nCounter Low RNA Input Amplification Kit (NanoString Technologies, Seattle, WA, USA), as previously described [38]. The PanCancer Pathways panel, containing 770 genes, was used to analyze EV-preamplified cDNA, according to manufacturer’s protocol. Hybridization step was performed for 18 h at 65 °C.

The geometric mean plus twice the standard deviation of the negative control probes was used as background for data normalization. Then, the total signal from all genes above the background was established as a criterion for sample normalization. Subsequently, to be more restrictive, an expression threshold value was added, based on the geometric mean plus twice the standard deviation of the background signal. All the genes that showed expression above this threshold in at least one of the groups (control; BPH or mPC; plasma or urine) were selected. For differential gene expression analysis, genes with fold change values greater than 2 or less than -2 were selected and analyzed by Ingenuity Pathway Analysis (IPA, Qiagen) software to determine the main signaling pathways in which they were involved. GraphPad Prism 6.00 software (GraphPad Software Inc., San Diego, CA, USA) was used to design the figures.

**Figure 1:**
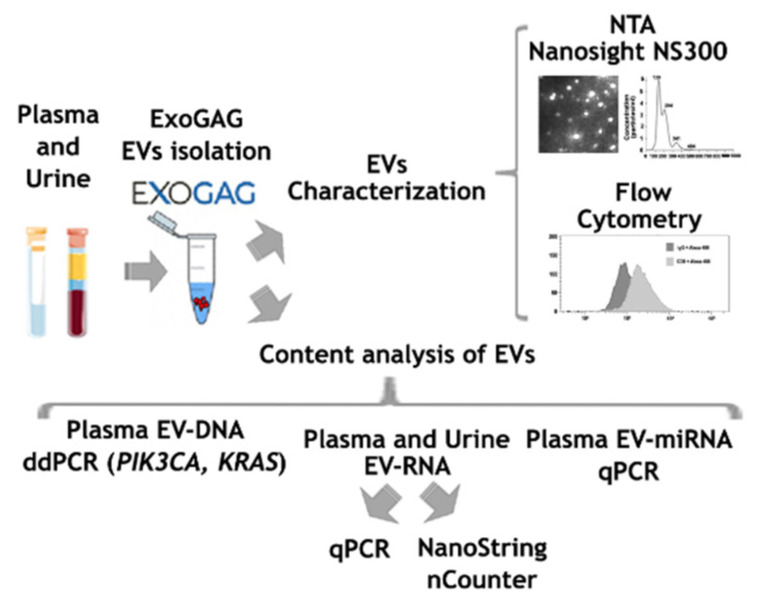
Scheme showing the steps followed for the characterization of EVs isolated with ExoGAG from plasma and urine samples.

As a summary, an outline showing all the steps included in the study is presented in Figure 1.

## 3. Results

### 3.1. Isolation and Characterization of EVs from Plasma and Urine Samples Using ExoGAG

EVs were purified from plasma and urine samples from healthy donors using ExoGAG technology, as previously described [33]. Briefly, samples were incubated with ExoGAG reagent for 5 min and then centrifuged at 3000× *g* for 15 min at 4 °C. The pellet containing EVs was resuspended in PBS and characterized by NTA and flow cytometry.

#### 3.1.1. Characterization of EVs from Plasma Samples

**Figure 2:**
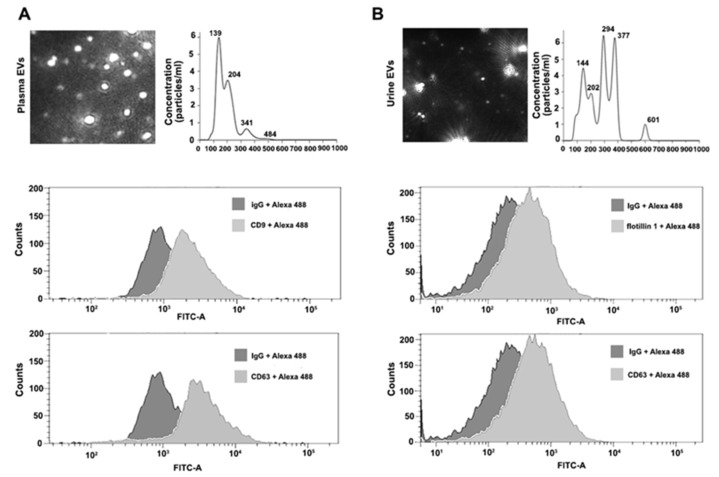
Characterization of plasma and urine EVs isolated by ExoGAG technology. (**A**) Representative image of NTA profile showing concentration (particles/mL) and size (nm) of plasma EVs (upper panel). Representative profiles of flow cytometry assays for plasma EVs. Alexa 488 fluorescence was used as secondary antibody and non-specific IgG as negative control of fluorescence. Positive CD9 and CD63 labelling is represented by a displacement of fluorescence peak curve (lower panels). (**B**) NTA profile of harvested urine EVs expressed as concentration (particles/mL) and size (nm) (upper panel). Flow cytometry analysis of urine EVs resulted in a positive flotillin 1 and CD63 labelling represented by a displacement of fluorescence peak curve (lower panels).

EVs isolated using ExoGAG from 50 μL of plasma from healthy donors (control) were resuspended in 1 mL of PBS and loaded into the NTA chamber, providing information of particle concentration and size according to their Brownian motion. EVs isolated from control plasma samples showed a particle profile around 190 nm on average and a concentration of 6.19 × 10^8^ particles/mL (Figure 2A, upper panel). For flow cytometry analysis, 100 μL of control plasma samples were used to purify EVs by ExoGAG. The pellet containing EVs was resuspended in 200 μL of PBS and analyzed by flow cytometry as described in Materials and Methods, revealing the presence of the characteristic EV-associated biomarkers CD9 and CD63, represented by a displacement of the fluorescence curve in comparison to the IgG fluorescence profile used as negative control (Figure 2A, lower panels).

#### 3.1.2. Characterization of EVs from Urine Samples

Prior to the isolation of EVs by ExoGAG, urine samples were depleted of uromodulin (see Materials and Methods) for NTA characterization and flow cytometry analysis. Briefly, EVs from 3 mL of control urine samples were isolated by ExoGAG and resuspended in 1 mL of PBS. The concentration of particles and size distribution were analyzed by NTA, showing a heterogeneous particle size population compared to the profile of plasma samples, and with intensity peaks ranging from 144 to 601 nm (Figure 2B, upper panel). The mean peak of detected particles was 272.7 nm and the concentration of EVs was 1.1 × 10^10^ particles/mL. In parallel, 3 mL of control urine samples were incubated with ExoGAG and the purified EVs were resuspended in 200 μL of PBS to perform flow cytometry analysis. As previously described for plasma samples, the results showed a specific EV’s positive labelling for the EV markers flotillin 1 and CD63, represented by a displacement of the fluorescence curve compared to the fluorescence peak from the negative control IgG (Figure 2B, lower panels).

### 3.2. Point Mutation Detection by Droplet Digital PCR (ddPCR) in DNA from Plasma EVs Isolated by ExoGAG

**Figure 3:**
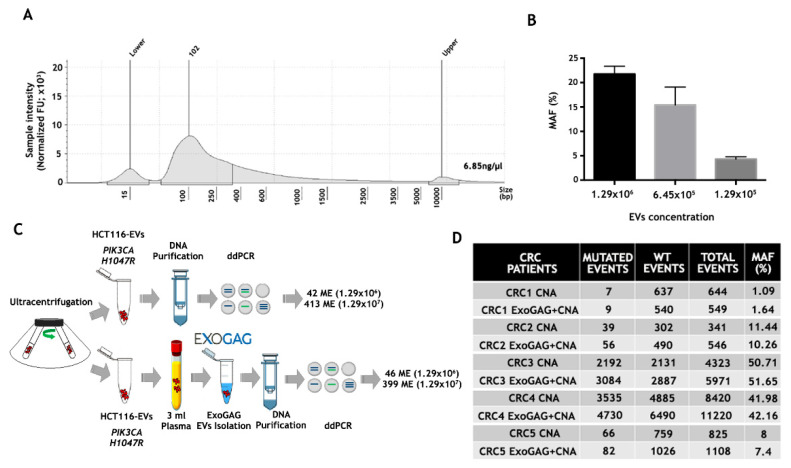
Analysis of point mutations by droplet digital PCR (ddPCR) in DNA from plasma EVs isolated by ExoGAG. (**A**) Quantification and fragment distribution of the EV-DNA purified from EVs isolated with ExoGAG from a plasma control sample analyzed by TapeStation4200 using High Sensitivity D5000 ScreenTapes Kit. (**B**) Histogram showing the percentage of MAF (% MAF) for the *PIK3CA H1047R* mutation in EVs isolated by ExoGAG from healthy donor (control) plasma samples spiked with 1.29 × 10^6^, 6.45 × 10^5^ and 1.29 × 10^5^ EVs from the HCT116 cell line and precipitated with ExoGAG. Mean (SD) of two independent assays. (**C**) Performance of ExoGAG technology for the isolation of EVs, comparing the number of mutated events for *PIK3CA H1047R* in two concentrations of HCT116-EVs isolated by ultracentrifugation, and when the same concentrations of EVs are added to control plasma samples and precipitated with ExoGAG. (**D**) Number of mutated, wild-type (WT), total events and percentage of MAF (%), corresponding to the DNA samples of EVs isolated by ExoGAG (ExoGAG + CNA) from plasma samples of five CRC patients harboring *KRAS G12V* mutation and the same plasma samples processed by standard methodology (CNA).

Once EVs from control plasma samples were isolated by ExoGAG and characterized, the genetic material contained in the purified EVs was studied. To this end, EVs were isolated from 3 mL of control plasma sample by ExoGAG technology, and the EV-DNA was extracted using the QIAamp Circulating Nucleic Acid Kit (CNA), initially designed for the purification of circulating free DNA, RNA and miRNA from samples containing low concentrations of mostly fragmented DNA and RNA. The quality and concentration of EV-DNA were analyzed by TapeStation 4200 using a High Sensitivity D5000 ScreenTapes Kit (Figure 3A), showing a concentration of 6.85 ng/µL and a fragment distribution between 100–10000 base pairs (bp) with a peak at 102 bp.

The efficiency of both the isolation of EVs from plasma using the ExoGAG reagent and the detection of point mutations in the EV-DNA contained in these EVs was analyzed by ddPCR. In order to analyze the sensitivity in the detection of mutations in the EV-DNA of EVs isolated with ExoGAG, a spiking of 3 mL of control plasma sample with 1.29 × 10^6^, 6.45 × 10^5^ and 1.29 × 10^5^ EVs from the colorectal HCT116 cell line isolated by ultracentrifugation was performed; this was also carried out in order to detect the *PIK3CA H1047R* mutation present in this human colorectal cancer cell line [39]. The samples were processed with ExoGAG as described previously, and the EV-DNA was extracted with the CNA Kit. The analysis of the *PIK3CA H1047R* mutation by ddPCR resulted in a percentage of MAF of 21.77%, 15.41% and 4.29%, respectively (Figure 3B), evidencing the linearity and high sensitivity of *PIK3CA H1047R* mutation detection by ddPCR in the EV-DNA.

In order to determine the ExoGAG-based EVs’ isolation efficiency, two concentrations of EVs from the HCT116 line, previously isolated by untracentrifugation, were added to 3 mL of control plasma. In parallel, the same amount of EVs, collected directly from the ultracentrifugation, were analyzed. The ddPCR analysis showed a similar number of mutated events in EV-HCT116 isolated by ultracentrifugation (42 and 413, respectively) and those added to the plasma and isolated by ExoGAG (46 and 399, respectively) (Figure 3C). These results showed an optimal performance in the isolation of EVs and confirmed the efficiency of point mutation detection by ddPCR in EV-DNA using ExoGAG technology. 

Finally, with the aim of translating these results to real clinical samples, the detection of the *KRAS G12V* mutation was evaluated in EVs isolated with ExoGAG technology from plasma samples of colorectal cancer (CRC) patients carrying the mutation (previously determined by Beaming technology). Briefly, EVs were isolated from 3 mL of the CRC plasma samples using ExoGAG technology, and the EV-DNA was purified using the CNA Kit as previously described. In parallel, DNA from the same volume of plasma was isolated with the CNA Kit as standard technology. ddPCR comparative analysis showed a similar percentage of MAF in both procedures (Figure 3D). However, the number of events detected in the DNA of EVs isolated with ExoGAG (ExoGAG + CNA) was higher in most of the cases analyzed compared to standard technology (CNA). This increased sensitivity in detecting mutated events, while maintaining the MAF percentage, shows the clinical value of ExoGAG technology, mainly in the detection of mutations that are in low prevalence.

### 3.3. Optimization of Plasma and Urine EV-RNA Purification Methodologies

**Figure 4:**
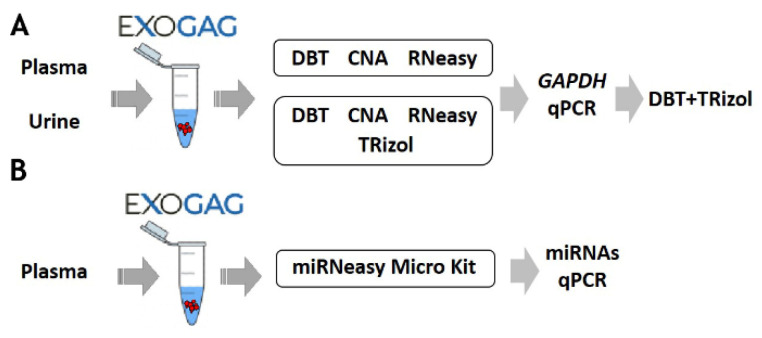
Schematic of the protocol for optimization of (**A**) RNA and (**B**) miRNA purification methodologies of EVs isolated with ExoGAG from plasma and urine.

Once the efficiency and sensitivity of point mutation detection in EV-DNA isolated using ExoGAG technology were confirmed, the possibility to study the RNA cargo in EVs was explored. For this purpose, the most efficient method to purify EV-RNA after ExoGAG purification from control plasma samples was assessed, and three commercial RNA extraction methods were comparatively analyzed and selected based on their ability to purify total RNA from low concentration samples (Figure 4; see Supplementary Material S1). To evaluate the EV-RNA yield with the three different RNA extraction methods, a RT-q-PCR of the *GAPDH* housekeeping gene was performed. Comparatively, the most efficient method for EV-RNA extraction was found to be the DBT Kit with a previous TRIzol-chloroform lysis step (Figure S1), showing an optimal level of *GAPDH* expression compared to the other methodologies (Figure S2A).

Furthermore, the most efficient methodologies (DBT + TRIzol and CNA) were also compared to determine the extraction of EV-RNA from a control urine sample. As in plasma samples, the optimal *GAPDH* expression, plotted as 40-Cq mean, was observed when the DBT Kit with a previous TRIzol-chloroform lysis step was used (Figure 4 and Figure S2B). These results show the possibility of using ExoGAG for the isolation of EVs from plasma and urine and the subsequent analysis of their RNA content.

Additionally, as a proof of concept and in order to determine whether the EVs isolated with ExoGAG would allow further analysis of their miRNA content, a plasma sample was analyzed. After isolation of the EVs, the analysis of miRNAs was carried out by qPCR, following purification with the microRNeasy Micro Kit (Figure 4 and Figure S3).

### 3.4. EV-mRNA Analysis Using nCounter PanCancer Pathways Panel

**Figure 5:**
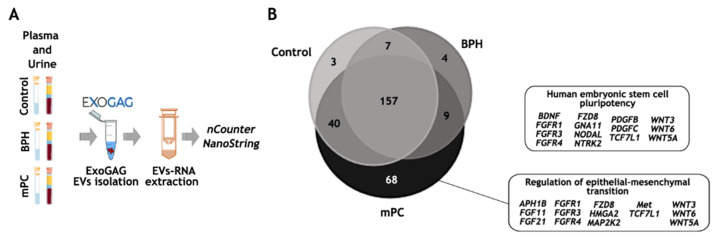
EV-mRNA analysis on the nCounter NanoString platform. (**A**) Workflow for EV-mRNA purification and analysis on the nCounter platform: EVs were isolated with ExoGAG technology from plasma and urine samples. EV-mRNA was purified using DBT Kit with a previous lysis step of TRIzol-chloroform. Next, a retro-transcription and a pre-amplification step were performed prior to hybridization with the Pancancer Pathways panel. (**B**) Total genes identified in control, BPH and mPC plasma EV samples represented in a Venn diagram showing 157 common genes and 3, 4 and 68 genes represented only in the control, BHP and mPC, respectively, once the threshold value was established. The main IPA signaling pathways represented by 68 genes expressed only in mPC plasma sample were related to the human embryonic stem cell pluripotency pathway and regulation of epithelia-mesenchymal transition pathway. This figure was produced using Servier Medical Art (https://smart.servier.com accessed on 10 October 2022) licensed under a Creative Commons Attribution 3.0 Generic License.

The efficiency of RNA extraction and quality upon purification of the EVs with ExoGAG technology permitted us to perform a feasibility pilot study on subsequent mRNA analysis using the NanoString nCounter platform that enumerates RNA targets via hybridization of sequence-specific fluorescent barcodes. For this purpose, the PanCancer Pathways panel containing 770 genes related to prostate cancer was chosen. EV-mRNA expression from three clinically relevant samples was analyzed: a healthy donor (Control), a benign prostatic hyperplasia (BPH) and a metastatic prostate cancer (mPC), and plasma and urine samples were also analyzed comparatively (Figure 5A). Briefly, 6 mL of plasma was used to isolate EVs by ExoGAG technology. Subsequently, EV-RNA was extracted using the DBT Kit, adding a previous TRIzol-chloroform lysis step, as previously described. After retro-transciption, a pre-amplification step was performed using the nCounter Low RNA Input Amplification Kit with primers targeting the genes of the PanCancer Pathways panel, selecting 10 cycles, according to the previous work by Bracht et al. [38]. Finally, pre-amplified cDNA was analyzed by nCounter using the PanCancer Pathways panel.

As expected, the number of transcripts identified above the background in the EV-mRNA mPC plasma sample was higher (457 genes) compared to those identified in the EV-mRNA control plasma sample (271 genes) and in the EV-mRNA BPH sample (198 genes) (Tables S1–S3). To be more restrictive, and once the genes above the background were selected, only those genes in at least one of the groups whose values were higher than the threshold were taken into account, obtaining a final list of 288 genes (see Material and Methods). Among these, 157 genes were commonly expressed in the 3 study groups, while 3, 4 and 68 genes were represented only in the control, the BHP and the mPC samples, respectively (Figure 5B). Of note, the ingenuity pathway analysis (IPA) focused on the 68 genes exclusively expressed in EV-mRNA from the mPC plasma sample identified pluripotency and self-renewal, on the one hand, and epithelial-to-mesenchymal (EMT) features, on the other, as the two main signaling pathways involved in the metastasis process in prostate cancer.

**Figure 6:**
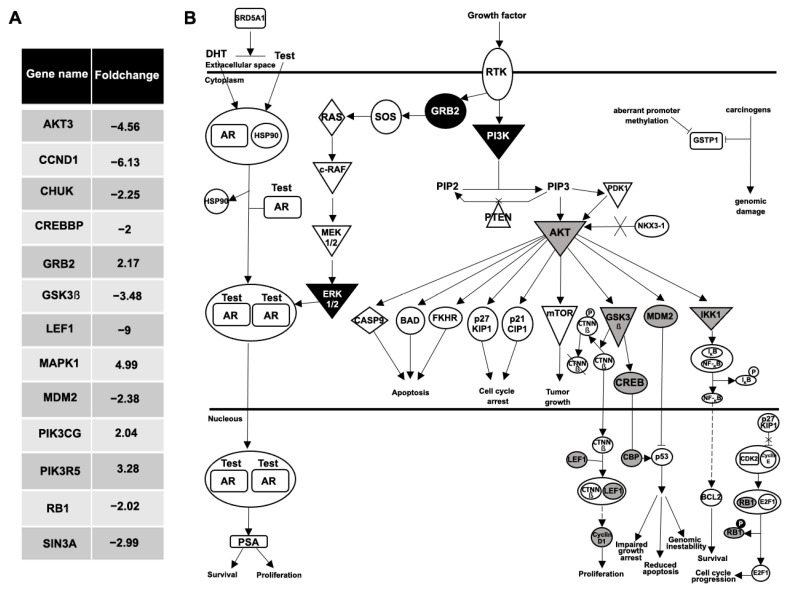
Analysis of prostate cancer pathway. (**A**) Fold change values of 13 differentially expressed genes in prostate cancer pathway between the plasma EVs from the BPH and the mPC samples. (**B**) Ingenuity pathway analysis network of prostate cancer signaling: nine genes were downregulated (AKT3, CCND1, CHUK, CREBBP, GSK3β, LEF1, MDM2, RB1 and SIN3A; represented in gray color), and four genes were upregulated (GRB2, MAPK1, PIK3CG, PIK3R5; represented in black color), in EV-mRNA from mPC plasma sample compared to EV-mRNA from BPH plasma sample.

Also of interest, when a gene-expression comparative analysis between the plasma EVs from the BPH and the mPC samples was performed, 48 genes were differentially expressed with at least a two-fold increase or decrease (Figure 6A). The IPA software analysis showed the *prostate cancer network* and *molecular mechanism of cancer* as the main signaling pathways related to these differentially expressed genes. Focusing on the analysis of the prostate cancer pathway, genes upregulated (GRB2, MAPK1, PIK3CG and PIK3R5; black color) and downregulated (AKT3, CCND1, CHUK, CREBBP, GSK3β, LEF1, MDM2, RB1 and SIN3A; gray color) in EV-mRNA from an mPC plasma sample compared to EV-mRNA from BPH plasma sample are illustrated in Figure 6B. 

**Figure 7:**
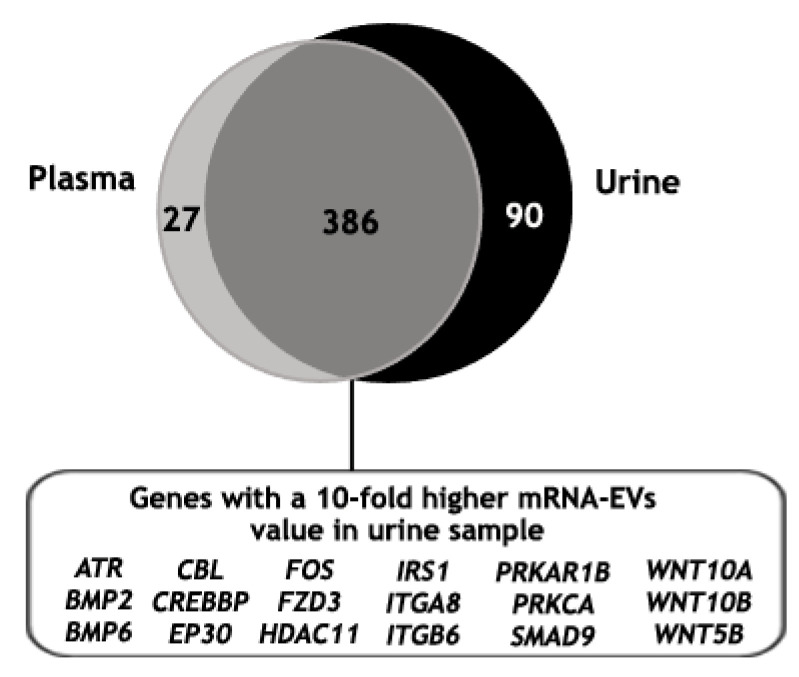
Identified genes above the threshold value in plasma and urine mRNA-EVs samples from an mPC patient, represented in a Venn diagram showing 386 common genes, of which 20% resulted in a 10-fold higher expression in EV-mRNA from mPC urine sample compared to the EV-mRNA mPC plasma sample. This set of genes was involved mainly in molecular mechanism of cancer pathways where ATR, BMP2, BMP6, CBL, CREBBP, EP30, FOS, FZD3, HDAC11, IRS1, ITGA8, ITGB6, PRKAR1B, PRKCA, SMAD9, WNT10A, WNT10B and WNT5B were represented.

Finally, the mRNA gene expression analysis of EVs isolated from the urine of the same mPC patient was performed, identifying 489 genes above the background (Table S4). Then, the mRNA gene expression of EVs isolated with ExoGAG from the plasma and the urine samples of this patient was compared. For this, only those genes with values higher than the threshold in at least one of their groups were taken into account (see Material and Methods). As expected, the majority of the identified genes were commonly expressed in both samples (386 genes); in addition, 27 and 90 genes were identified exclusively in the plasma and in the urine samples, respectively (Figure 7). Furthermore, when the 386 genes in common were analyzed, 37% of the genes showed a 5- to 10-fold higher expression in the EV-mRNA from mPC urine sample compared to the plasma sample and 20% showed a 10-fold higher expression. This 20% of differentially expressed genes were involved mainly in molecular mechanisms of cancer signaling when an ingenuity pathway analysis was performed (Figure 7).

All these results demonstrated the feasibility of EV-mRNA expression analysis in EVs isolated by ExoGAG both from plasma and urine samples. Moreover, we demonstrated the potential clinical value of EV-mRNA analysis for the identification of molecular pathways associated with a specific clinical process, in this case, with the evolution of a prostate lesion from a benign hyperplasia condition into a metastatic cancer process, linked to the acquisition of pluripotency and EMT features. It should be mentioned that this is a pilot study using a limited number of samples, with the aim of assessing the feasibility of a methodology based on the mRNA gene expression of EVs purified with the ExoGAG technology from plasma and urine samples.

## 4. Discussion

The advent of liquid biopsy and the analysis of biomarkers in biological fluids has overcome the limitations of traditional tissue biopsies such as accessibility, invasiveness, repeatability, heterogeneity and dynamic tumor evolution. Recently, the two most developed biomarkers in liquid biopsy—circulating tumor cells (CTC) and cell-free tumor DNA (ctDNA)—have been joined by a powerful tool, the analysis of extracellular vesicles (EVs). The fact that these vesicles are released by living cells and not by cells undergoing necrosis or apoptosis, as with ctDNA, in addition to the protection that their membranes provides to their content (including proteins, lipids, DNA, mRNA or microRNAs, etc.) are additional advantages [40,41,42]. 

Due to their important role in tumor progression, participating in angiogenesis processes, modulation of immune response and in the establishment of premetastatic niche [43,44,45], the study of EVs in the field of cancer is one of the most widespread. However, their involvement in other pathological conditions has also been described. For example, there is evidence of their involvement in thrombosis [46], in reproductive [47] and neurodegenerative disorders [48,49] or in transplant rejection [50]. 

Despite their potential, one of the main limitations in the clinical implementation of EVs is the absence of fast, efficient and high-degree purity isolation technologies that allow for scaling, automation and translation into clinical routine. To overcome this barrier, we have developed ExoGAG, a commercially available technology that, through the use of a precipitating agent, enables the rapid and efficient isolation of EVs from different biological fluids, with the only requirement of a conventional centrifuge. In our previous work, we demonstrated that ExoGAG is efficient in the isolation of EVs from plasma samples of endometrial cancer patients and identified ANXA2 protein as a possible EV-based prognostic biomarker in liquid biopsy [33]. 

In this pilot study, the DNA and RNA content of a small number of EV samples isolated with ExoGAG was studied, with the intention of demonstrating that our technology is compatible with downstream genetic analysis.

In a first step, EVs isolated from plasma and urine samples using ExoGAG were characterized to verify that the pellet was enriched in EVs. As there is currently no unique, specific marker, a combination of characterization methods was carried out, including determination of size by NTA Nanosight and expression of EV-enriched proteins by flow cytometry, such as tetraspanins CD9 and CD63 and Flotillin 1, involved in membrane transport and fusion. A volume of only 200 μL was used to carry out these determinations, which is an advantage when there is limited access to samples. The EVs from the urine sample, despite being positive for the previously mentioned markers, showed a relatively heterogeneous population of extracellular vesicles, with a larger mean size, around 272 nm, probably due to the variable nature of urine (pH), but similar to that described with other isolation methods [51].

Once the efficacy of EV isolation was confirmed, a sensitivity analysis was carried out, considering that, to date, numerous publications have described the presence of point mutations in the DNA contained in EVs (EV-DNA) and their potential use as diagnostic and prognostic biomarkers [52,53]. Although it has been reported that most of the exosomal DNA is located on the outer surface of the vesicles, it has been shown that the quality and size of the DNA contained inside the vesicles is higher than that of free circulating DNA (cfDNA), which implies an advantage for further analysis such as NGS [54]. Comparative analyses, both at the level of artificial samples (plasma to which exosomes carrying a known mutation were added), and at the level of real patient samples, showed that ExoGAG had optimal exosome isolation efficiency, as well as high sensitivity in the detection of point mutations (*PIK3CA, KRAS*). 

In fact, a higher detection capacity of the events number in the ExoGAG-treated samples was detected, which would be particularly beneficial in the earlier stages of disease where ctDNA levels are very low. The results support the use of ExoGAG technology in the isolation of EVs from plasma samples and the subsequent detection of clinically valuable EV-DNA alterations, where an increase in sensitivity compared with ctDNA alone has been reported [55]. In addition, the technology would allow an increase in the starting sample volume to improve detection sensitivity in the case of patients with low tumor burden.

As for the RNA contained within the EVs (EV-RNA), it has been reported to be more stable in circulation, compared to cellular RNAs [56,57], and sequencing studies have shown that all types of RNAs exist within EVs (mRNAs, lncRNA, ribosomal RNA, tRNA and noncoding RNAs). This, together with its important biological functions, implies that the analysis and characterization of EV-RNA content represents a promising source of cancer biomarkers. Therefore, in this work, different EV-RNA isolation methods for EVs purified by ExoGAG from plasma and urine samples were explored with the aim of subsequently performing a 770-gene expression profiling based on the nCounter Pancancer Panel by NanoString. In general, an increase in the RNA yield, measured by *GAPDH* amplification by quantitative PCR, was obtained by adding a TRIzol lysis step to the standard protocols, mainly in the selected kit, the DNeasy Blood and Tissue Kit (DBT, Qiagen). 

This small study involved the analysis of EV-mRNA contained in EVs isolated with ExoGAG from plasma and urine samples from a healthy donor (control), a patient with BPH and a patient with mPC. IPA analysis revealed that one of the main signaling pathways in which these genes were involved included the prostate cancer pathway. The profile of EV-mRNA detected in the mPC plasma sample, with the expression of 68 unique genes, showed two main signaling pathways in the IPA analysis, the human embryonic stem cell pluripotency pathway and regulation of epithelial-mesenchymal transition (EMT) pathway. These results were consistent with data from other authors describing the involvement of EMT in the regulation of prostate cancer stem cells, cancer progression and metastasis formation [58] as well as the content of the EVs in the promotion of stemness and EMT of low aggressive prostate cancer cells [59]. More specifically, it has been described that the upregulation of Frizzled 8 (FZD8), a Wnt family receptor expressed in the EV-mRNA from mPC plasma samples, promotes invasion and stem cell-like phenotypes in vitro through the activation of Wnt/β-catenin signaling, promoting prostate cancer bone metastasis [60]. The activation of this pathway and the metastasis process is also favored by WNT5A-mediated signaling [61]. Moreover, activation of FGFR1, also expressed in the EV-mRNA mPC sample, was described as an important factor to initiate carcinogenesis and EMT in prostate cancer [62]. All these data suggest that mRNA molecules were selectively incorporated into EVs.

To explore the gene expression profile of urine EVs and to confirm that ExoGAG-based isolation allows this subsequent analysis, a sample of urine EV-mRNA from the same mPC patient was included in the nCounter analysis. In total, 20% of the genes common to both plasma and urine samples showed a 10-fold higher expression in the EV-mRNA from mPC urine sample. These results showed that urine samples could represent an ideal source of liquid biopsy, by the possibility of obtaining large amounts of sample in a noninvasive way, to develop gene expression EV-mRNA analysis. In this regard, different authors have described differences between the profile of free mRNAs and miRNAs in plasma and the content of EVs in urine samples, suggesting targeted encapsulation [63] and making them potential biomarkers for diagnostic applications, not only in the field of oncology. In fact, exosomes from distant sites, such as the liver, have also been found in the urine [64]. Additionally, a recent study revealed the great potential of urine exoDNA to detect somatic mutations, such as *KRAS*, compared to exoDNA from serum samples in bladder cancer [65]. In fact, urinary EVs are a promising source of tumor DNA in urological malignancies, due to its direct contact with the tumor. 

This pilot trial demonstrated the potential of ExoGAG technology to perform subsequent EV-mRNA gene expression analysis using nCounter NanoString technology, not only from plasma samples but also from urine. The information that could be obtained from this type of analysis would allow in vitro and in vivo functional studies, and is of great relevance in liquid biopsy. In this regard, it has been described that EV-RNA improves the sensitivity of detection of genetic alterations when combined with classical ctDNA analysis [66]. One of the main challenges to improve in future studies would be to confirm EVs’ housekeeping genes, which would allow better normalization between samples. In this case, the total signal from all genes above the background was established as criteria for sample normalization. 

Taking into account the promising results obtained by analyzing EV-mRNA, another possible application of ExoGAG-isolated EVs was explored and the expression of miRNAs in plasma EVs was analyzed. The study highlighted the potential to detect these EV-miRNAs from a volume as little as 125 µL, important when there is a limitation in the amount of sample available. Several studies have demonstrated the usefulness of EV-miRNA as diagnostic biomarkers in different tumors. Thus, increased levels of exosomal miR-23b-3p, miR-10b-5p and miR-21-5p have been associated with poor overall survival (OS) in plasma samples from NSCLC patients [67]. In CRC, exosomal miR-21 and miR-19b have showed higher sensitivity and specificity compared to the routine clinical biomarker CEA [40]. Moreover, increased levels of EVs’ miR-21 could distinguish esophageal squamous cell cancer (ESCC) patients from benign diseases, being this EV miRNA also correlated with cancer progression indicating that EVs miR-21 could be a diagnostic and prognostic biomarker [68]. In addition, increased levels of miR-1290 and miR-375 have been found in EVs in plasma from advanced prostate cancer patients and these levels correlated with a decline in OS [69]. High levels of exosomal miR-1246 correlated also with poor prognosis in this type of cancer [70]. In this pilot trial, the expression of miR-491, which plays a role as tumor suppressor gene in numerous types of cancer [71,72,73], and miR-16, which is associated with tumor progression and could be a potential biomarker for human cancer diagnosis [74,75,76], were analyzed by quantitative PCR. 

One of the main limitations of the study is associated with the ExoGAG-based exosome isolation method. As it is a non-specific method, i.e., based on a tumor biomarker, a heterogeneous population of exosomes from different origins, not only of tumor origin, is recovered, although their levels have been shown to be elevated in cancer. Therefore, it is not possible to speak of a method of isolation of tumor-derived exosomes. On the other hand, the isolation method itself opens up the possibility of screening for new biomarkers enriched in tumor exosomes and the extension of its potential to the search for new targets present in exosomes of liquid biopsy samples from other types of pathologies. 

As with other LB sources (CTCs, ctDNA, RNA), tumor-derived exosomes are relatively rare in biological fluids compared to non-tumoral exosomes, which could hamper detection capabilities [77] and may lead to false positive and false negative results. It is therefore necessary to carry out studies in which the number of samples included is relevant. In this sense, another limitation of this study is the reduced number of samples from healthy, BHP and mPC patients. The study was intended as a proof of concept to determine whether EVs isolated with ExoGAG technology would allow further analysis of their DNA, mRNA and miRNA content, as well as to confirm the efficiency and sensitivity of the EV isolation technology. These objectives have been met in this small study, but the number of samples should be greatly increased to reach robust conclusions and to validate their performance and clinical utility.

## 5. Conclusions

This proof of concept study indicated that ExoGAG allows the user-friendly isolation of EVs from plasma and urine samples in an efficient and sensitive manner. In addition, isolated EVs allow for downstream applications, such as point mutation detection by ddPCR, mRNA and miRNA, analysis by qPCR and gene expression analysis by nCounter Nanostring. Although focused on cancer patients’ samples, these results provide evidence for the potential value of EVs as noninvasive biomarkers for diagnosis, prognosis and treatment response monitoring.

## Data Availability

The data presented in this study are available in Supplementary Material.

## Supplementary Materials

The following supporting information can be downloaded at: https://www.mdpi.com/article/10.3390/biomedicines11020404/s1, Figure S1: Schematic image of EV-RNA extraction method using DNeasy Blood and Tissue Kit (DBT) with or without a previous TRIzol-chloroform lysis step title, Figure S2: Plasma and urine EV-mRNA analysis by RT-q-PCR and TapeStation after the isolation of EVs using ExoGAG, Figure S3: Plasma EV-miRNA analysis by RT-q-PCR, Table S1: Transcripts identified in EV-mRNA from mPC plasma sample above the background, Table S2: Transcripts identified in EV-mRNA from control plasma sample above the background, Table S3: Transcripts identified in EV-mRNA from BPH plasma sample above the background, Table S4: Transcripts identified in EV-mRNA from mPC urine sample above the background.
