# Supplementary material for: Extracellular Vesicles’ Genetic Cargo as Noninvasive Biomarkers in Cancer: A Pilot Study Using ExoGAG Technology
Source: Biomedicines. 2023 Jan 30;11(2):404. doi: 10.3390/biomedicines11020404 (PMC9953104; doi:10.3390/biomedicines11020404)
Supplement: Supplementary file 1 [file biomedicines-11-00404-s001.zip › biomedicines-2129504-supplementary.pdf]

Supplementary Material S1.

EVs were purified by ExoGAG from 3ml of plasma and urine samples. Total EV-RNA was extracted using three commercial kits: QIAamp Circulating Nucleic Acid kit (CNA) (Qiagen, Hilden, Germany) and RNeasy Mini Kit (Qiagen, Hilden, Germany), according to the manufacturer's instructions and the DNeasy Blood and Tissue Kit (DBT) (Qiagen, Hilden, Germany) with some protocol modifications (see Figure S1). In parallel, these three kits were compared with the addition of a previous lysis step with TRIzol (TRIzol™ Reagent, 15596018, Thermofisher Scientific, Van Allen Way, CA, USA) and chloroform (J.T Baker, Gliwice, Poland). 750µl of TRIzol for every 500µl of sample were added, mixed thoroughly and incubated at room temperature (RT) for 5 min. Then, 200µl of chloroform for every 500µl of sample were added, mixed thoroughly and incubated at RT for 5min. The aqueous upper phase containing the RNA was collected after centrifugation at 12,000xg for 15min at 4°C and then each protocol was followed according to the instruc-tions. Treatment with DNase I (79254, Qiagen, Hilden, Germany) was carried out, ac-cording to manufacturer's protocol.

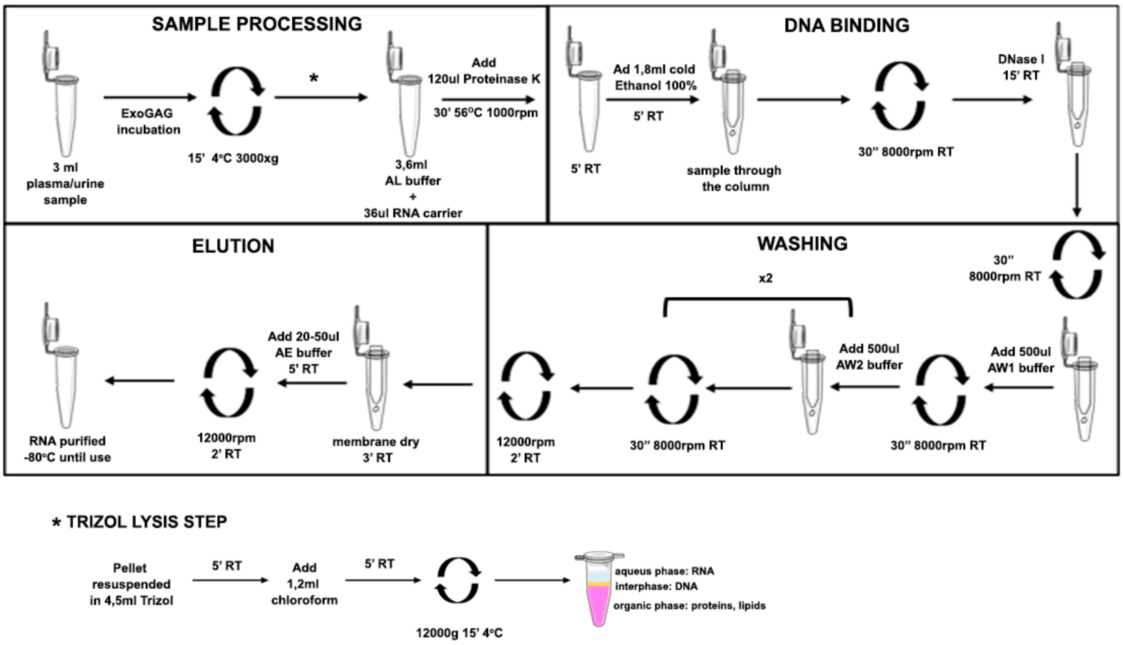

Figure S1. Schematic image of EV-RNA extraction method using DNeasy Blood and Tissue kit (DBT) with or without a previous TRIzol-chloroform lysis step. EVs were isolated from 3ml of plasma and urine samples using ExoGAG according to the manufacturer's instructions. 3,6ml of AL buffer with 36 µl RNA carrier were added and mixed thoroughly by vortexing. 120µl Proteinase K were added. The sample was mixed thoroughly by vortex, and incubated at 56°C, 30', 1000rpm. An incubate for 5min at room temperature (RT) and spin was performed. Then a 1,8ml cold ethanol (100%) were added and mixed by inverting tubes slowly. An incubation for 5min at RT and spin was performed. The mixture into the DNeasy Mini spin column was pipeted and placed in a 2ml collection tube. The sample was centrifugated at 8000rpm for 30s. Flow-through was discarded. An incubation with DNasa I for 15min at RT and a centrifugation for 30s at 8000 rpm were performed. Flow-through was discarded. The DNeasy Mini spin column was placed in a new 2ml collection tube. 500µl of Buffer AW1 was added and a centrifugation for 3 s at 8000rpm was performed. Flow-through was discarded. The DNeasy Mini spin column was placed in a new 2ml collection tube. 500µl Buffer AW2 was added and a centrifugation for 30s at 8000rpm was performed. Flow-through was discarded. This step was repeated once. A centrifugation at 12,000rpm 2min was performed. An incubate for 3min at RT with the DNeasy Mini spin column cap open for the ethanol to evaporate was developed. DNeasy Mini spin

column was placed in a clean tube and 20-50µl Buffer AE were pipeted directly into the DNeasy membrane. An incubation for 5min at RT and a centrifugation for 2min at 12,000rpm to elute were performed.

* TRIzol LYSIS STEP: EVs pellet was resuspended in 4,5ml of TRIzol. A vortex and an incubation for 5min at RT were performed. 1,2ml of chloroform was added and a vortex and incubation for 5min at RT was performed. The sample was centrifugated at 12,000x*g*, 4ºC, 15min. A centrifugation was performed to separate the mixture into 3 phases: a organic phase containing the protein, an interphase, usually white, containing the DNA, a colorless upper aqueous phase containing RNA. The colorless upper aqueous phase was transferred to a new clean tube and the previously described protocol was continued. This figure was produced using Servier Medical Art (https: //smart.servier.com) licensed under a Creative Common Attribution 3.0 Generic Licence.

The results showed that CNA was the most efficient methodology to purify EV-RNA, presenting the maximal *GAPDH* expression signal plotted as 40-Cq mean (Figure S2A, grey bars). We further introduced a previous lysis step with TRIzol-chloroform to the three extraction methodologies to assess whether release of the EVs content represents a critical step for an improved EV-RNA extraction yield. As shown, the RNA extraction yield was increased when a previous lysis step with TRIzol-chloroform was introduced in the DBT and RNeasy Mini kits, showing an improved *GAPDH* expression signal represented as 40-Cq mean (Figure S2A, black bars). In the case of the CNA extraction method, no significant improvement in the EV-RNA yield was observed by the previous TRIzol-chloroform lysis treatment, suggesting that no additional release of the EVs content was achieved by an extra lysis step. Comparatively, the most efficient method for EV-RNA extraction was found to be the DBT kit with a previous TRIzol-chloroform lysis step, showing an optimal level of *GAPDH* expression compared to the other methodologies. Finally, the concentration and quality of the EV-RNA isolated using ExoGAG from plasma sample and purified with the selected RNA extraction method (DBT+TRIzol) were measured by TapeStation 4200, showing a concentration of 0.499 ng/ul (Figure S2A; right panel) with a fragment distribution between 200-6000 nucleotides (nt) with a peak around 500 nt.

Furthermore, the most efficient methodologies (DBT + TRIzol and CNA) were also compared to determine the extraction of EV-RNA from a control urine sample. As in plasma samples, the optimal *GAPDH* expression, plotted as 40-Cq mean, was observed when the DBT kit with a previous TRIzol-chloroform lysis step was used (Figure S2B, left panel). The quality and concentration of EV-RNA contained in the EVs isolated with ExoGAG from urine samples were also tested by TapeStation 4200, showing a concentration of 1.03ng/ul and a fragment distribution also between 200-6000 nucleotides (nt), with a peak around 700nm (Figure S2B; right panel).

These results show the possibility of using ExoGAG for the isolation of EVs from plasma and urine and the subsequent analysis of their RNA content.

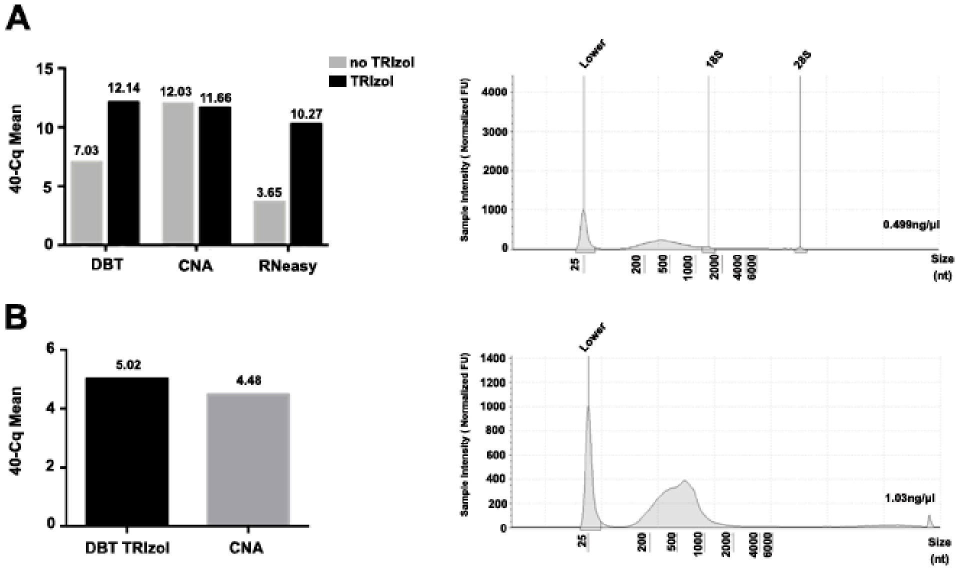

Figure S2. Plasma and urine EV-mRNA analysis by RT-q-PCR and TapeStation after the isolation of EVs using ExoGAG. (A) Comparative analysis of three RNA extraction methods: QIAamp Circulating Nucleic Acid kit (CNA), RNeasy Mini Kit (RNeasy) and DNeasy Blood and Tissue kit (DBT) with (black bars) or without (grey bars) a previous lysis step with TRIzol-chloroform represented by *GAPDH* gene expression in plasma EVs isolated by ExoGAG. *GAPDH* gene expression is represented as 40-Cq Mean (left panel). Quantification and fragment distribution of the EV-RNA purified by DBT+TRIzol from plasma control sample analyzed by TapeStation4200 using High Sensitivity RNA ScreenTapes kit (right panel) (B) Analysis of *GAPDH* gene expression in EV-RNA from urine EVs isolated with ExoGAG, comparing DBT kit with a previous lysis step of TRIzol-chloroform and CNA kit and plotted as 40-Cq mean (left panel). Quantification and fragment distribution of the EV-RNA purified by DBT+TRIzol from urine control sample analyzed by TapeStation4200 using High Sensitivity RNA ScreenTapes kit (right panel).

Figure S3. Plasma EV-miRNA analysis by RT-q-PCR. EVs were purified by ExoGAG from 125µl of control plasma samples. EV-miRNA was purified using miRNeasy Micro kit (Qiagen, Hilden, Germany) according to the manufacturer's protocol with DNase I (79254, Qiagen, Hilden, Germany) treatment. cDNA was synthesized using TaqMan MicroRNA Reverse Transcription Kit (Applied Biosystems, Thermo Fischer, Baltics, UAB), using a fixed volume of total RNA and following thermal-cycling conditions of 16°C for 3 min, 42°C for 30 min, 85°C for 5min. Then, cDNA was pre-amplified using TaqMan® PreAmp Master Mix (Applied Biosystems, Thermo Fischer, Foster City, CA, USA) with the following thermal-cycling conditions: 95°C for 10min, 55°C for 2min, 72°C for 2min, 12 cycles at 95°C for 15s and 60°C for 4min, then 99,9°C for 10min. RT-q-PCR analyses were conducted on QuantSudioTM 3 instrument (Thermofisher Fisher Scientific, Waltham, MA, USA). Amplification was carried out using the following conditions: 50°C for 2 min, 95°C for 10min; 40 cycles (95°C for 15 sec and 60°C for 1 min). miR-491 and miR-16 expression values were represented as 40-Cq Mean. Data were analysed with the Quantstudio™ Design & Analysis software, version 2.5.1 (Thermofisher Fisher Scientific, Waltham, MA, USA).

Table S1. Transcripts identified in EV-mRNA from mPC plasma sample above the background.

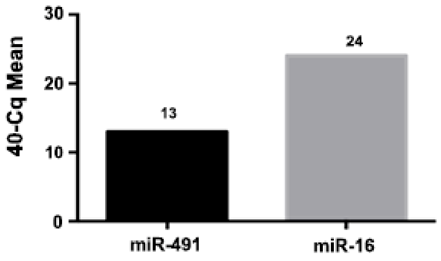

Gene	RefSEQ	Gene	RefSEQ	Gene	RefSEQ
**RPS27A**	NM_002954.5	**COL2A1**	NM_001844.4	**PAK7**	NM_177990.1
**H3F3A**	NM_002107.3	**GNAS**	NM_080425.1	**CEBPA**	NM_004364.2
**SKP1**	NM_170679.2	**MYD88**	NM_002468.3	**PDGFB**	NM_033016.2

**RBX1**	NM_014248.2	**PLA2G4C**	NM_003706.2	**SMARCA4**	NM_003072.3
**B2M**	NM_004048.2	**BCL2L1**	NM_138578.1	**CDC14B**	NM_003671.3
**CEBPE**	NM_001805.2	**MAP2K1**	NM_002755.2	**TBL1XR1**	NM_024665.4
**GADD45A**	NM_001924.2	**SETD2**	NM_014159.6	**CDKN2D**	NM_001800.3
**GZMB**	NM_004131.3	**DUSP8**	NM_004420.2	**IL1B**	NM_000576.2
**MYB**	NM_005375.2	**TPO**	NM_175722.1	**TCF7L1**	NM_031283.1
**PTEN**	NM_000314.3	**VHL**	NM_000551.2	**CAPN2**	NM_001748.4
**RHOA**	NM_001664.2	**PPP2CB**	NM_001009552.1	**FOS**	NM_005252.2
**HHEX**	NM_002729.4	**IFNA17**	NM_021268.2	**HMGA2**	NM_003484.1
**RAC2**	NM_002872.3	**RUNX1**	NM_001754.4	**PBX3**	NM_006195.5
**TFDP1**	NM_007111.4	**CD40**	NM_001250.4	**WNT6**	NM_006522.3
**ID2**	NM_002166.4	**AKT1**	NM_005163.2	**TNFSF10**	NM_003810.2
**UBB**	NM_018955.2	**NFKBIA**	NM_020529.1	**BRAF**	NM_004333.3
**HDAC2**	NM_001527.1	**PIM1**	NM_002648.2	**MYC**	NM_002467.3
**IL15**	NM_172174.1	**NCOR1**	NM_006311.3	**NODAL**	NM_018055.3
**SHC1**	NM_183001.4	**PIK3R5**	NM_001142633.1	**NR4A1**	NM_173157.1
**HIST1H3B**	NM_003537.3	**FOXO4**	NM_005938.2	**ACVR1B**	NM_004302.3
**RFC4**	NM_181573.2	**PCNA**	NM_002592.2	**EPOR**	NM_000121.2
**GNAQ**	NM_002072.2	**SOS2**	NM_006939.2	**XRCC4**	NM_003401.3
**RAD21**	NM_006265.2	**GRB2**	NM_002086.4	**HPGD**	NM_001145816.2
**H3F3C**	NM_001013699.2	**PBRM1**	NM_181042.3	**EFNA2**	NM_001405.3
**TCF3**	NM_003200.2	**PDGFA**	NM_002607.5	**CDKN1A**	NM_000389.2
**CCND3**	NM_001760.2	**DTX1**	NM_004416.2	**STMN1**	NM_203401.1
**CDKN1C**	NM_000076.2	**KAT2B**	NM_003884.3	**GNA11**	NM_002067.1
**STAT1**	NM_007315.2	**CASP8**	NM_001228.4	**BCL2**	NM_000657.2
**NBN**	NM_001024688.1	**CD19**	NM_001770.4	**ENDOG**	NM_004435.2
**RRAS2**	NM_001102669.2	**SOS1**	NM_005633.2	**FGFR1**	NM_015850.2
**HIST1H3H**	NM_003536.2	**HDAC5**	NM_005474.4	**GHR**	NM_000163.2
**TET2**	NM_001127208.2	**PRKAA2**	NM_006252.2	**CCND2**	NM_001759.2
**TGFB1**	NM_000660.3	**HDAC4**	NM_006037.3	**FANCC**	NM_000136.2
**DUSP6**	NM_001946.2	**XPA**	NM_000380.3	**NTRK2**	NM_001007097.1
**U2AF1**	NM_001025203.1	**PTPN11**	NM_002834.3	**SRSF2**	NM_003016.3
**PPP3R1**	NM_000945.3	**RELA**	NM_021975.3	**TIAM1**	NM_003253.2

**MAPK1**	NM_138957.2	**AKT2**	NM_001626.2	**MAP3K1**	NM_005921.1
**CYLD**	NM_015247.1	**JAK1**	NM_002227.1	**STK11**	NM_000455.4
**POLD4**	NM_021173.2	**BCL2A1**	NM_004049.2	**CDKN2B**	NM_004936.3
**IFNG**	NM_000619.2	**PRKAR2B**	NM_002736.2	**SPRY2**	NM_005842.2
**CDC7**	NM_003503.2	**RUNX1T1**	NM_004349.2	**APH1B**	NM_001145646.1
**NFE2L2**	NM_006164.3	**HDAC1**	NM_004964.2	**KMT2C**	NM_170606.2
**PIK3CG**	NM_002649.2	**GNG12**	NM_018841.3	**CCR7**	NM_001838.2
**CREB5**	NM_182898.2	**MAD2L2**	NM_001127325.1	**IGF1**	NM_000618.3
**PPP2R1A**	NM_014225.3	**BAX**	NM_138761.3	**NKD1**	NM_033119.3
**SMAD3**	NM_005902.3	**CREB3L4**	NM_130898.2	**PLA2G5**	NM_000929.2
**C19orf40**	NM_152266.3	**NR4A3**	NM_173198.1	**CHUK**	NM_001278.3
**FGF11**	NM_004112.2	**PRKCB**	NM_212535.1	**LEP**	NM_000230.2
**MAP2K4**	NM_003010.2	**RB1**	NM_000321.1	**MSH6**	NM_000179.1
**MAPK3**	NM_001040056.1	**DKK4**	NM_014420.2	**SYK**	NM_003177.3
**PRKACA**	NM_002730.3	**MAP3K8**	NM_005204.2	**CNTFR**	NM_147164.1
**SOCS3**	NM_003955.3	**HSPB1**	NM_001540.3	**FGF14**	NM_004115.3
**CDC25B**	NM_021873.2	**BRIP1**	NM_032043.1	**H2AFX**	NM_002105.2
**MUTYH**	NM_012222.2	**CD14**	NM_000591.2	**IDH2**	NM_002168.2
**HOXA11**	NM_005523.5	**DNMT3A**	NM_022552.3	**LEPR**	NM_001003679.1
**CCNA2**	NM_001237.2	**PDGFC**	NM_016205.1	**MAPK8**	NM_002750.2
**DTX4**	NM_015177.1	**POLR2J**	NM_006234.4	**MEN1**	NM_130802.2
**STAT3**	NM_139276.2	**BID**	NM_197966.1	**SOCS1**	NM_003745.1
**WHSC1L1**	NM_017778.2	**KLF4**	NM_004235.4	**SPRY1**	NM_005841.1
**CASP3**	NM_032991.2	**WHSC1**	NM_007331.1	**IL11**	NM_000641.2
**FGFR4**	NM_002011.3	**PML**	NM_002675.3	**RAC1**	NM_198829.1
**PIK3R1**	NM_181504.2	**COL5A1**	NM_000093.3	**STAT4**	NM_003151.2
**PRKDC**	NM_006904.6	**CTNNB1**	NM_001904.3	**GATA3**	NM_001002295.1
**FZD8**	NM_031866.1	**DUSP4**	NM_057158.2	**MAP3K13**	NM_004721.3
**MDC1**	NM_014641.2	**DUSP5**	NM_004419.3	**TP53**	NM_000546.2
**PLA2G4F**	NM_213600.2	**FAS**	NM_152876.1	**BNIP3**	NM_004052.2
**WNT3**	NM_030753.3	**GAS1**	NM_002048.2	**SPOP**	NM_001007226.1
**EFNA1**	NM_004428.2	**WNT5A**	NM_003392.3	**TNN**	NM_022093.1
**HIST1H3G**	NM_003534.2	**UBE2T**	NM_014176.3	**BRCA1**	NM_007305.2

**HSPA1A**	NM_005345.5	**EPO**	NM_000799.2	**CALML3**	NM_005185.2
**DAXX**	NM_001350.3	**FEN1**	NM_004111.4	**CDC14A**	NM_033313.2
**HMGA1**	NM_145904.1	**IL11RA**	NM_147162.1	**MCM7**	NM_182776.1
**COL11A1**	NM_001854.3	**NPM1**	NM_002520.5	**PRKACB**	NM_182948.2
**FGFR3**	NM_022965.2	**ITGA9**	NM_002207.2	**CCNE1**	NM_001238.1
**FLNA**	NM_001456.3	**LAT**	NM_001014987.1	**EZH2**	NM_004456.3
**MAP2K2**	NM_030662.2	**POLR2H**	NM_001278698.1	**ITGA7**	NM_002206.1
**PPP3CA**	NM_000944.4	**SMC1A**	NM_006306.2	**SP1**	NM_003109.1
**PPP3CB**	NM_001142354.1	**BDNF**	NM_170732.4	**THEM4**	NM_053055.4
**SIX1**	NM_005982.3	**KITLG**	NM_003994.4	**BMP2**	NM_001200.2
**FLNC**	NM_001127487.1	**SMAD2**	NM_001003652.1	**FGF9**	NM_002010.2
**RAF1**	NM_002880.2	**SMAD4**	NM_005359.3	**JAK2**	NM_004972.2
**TLX1**	NM_005521.3	**FGF21**	NM_019113.2	**MPO**	NM_000250.1
**CHEK2**	NM_007194.3	**HOXA9**	NM_152739.3	**PRMT8**	NM_019854.3
**SIRT4**	NM_012240.1	**IL7R**	NM_002185.2	**RAD50**	NM_005732.2
**ALKBH2**	NM_001001655.2	**MET**	NM_000245.2	**SOX17**	NM_022454.3
**ZAK**	NM_016653.2	**MDM2**	NM_006878.2	**ACVR1C**	NM_145259.2
**CCND1**	NM_053056.2	**HNF1A**	NM_000545.4	**ATRX**	NM_000489.3
**FGF23**	NM_020638.2	**MGMT**	NM_002412.3	**COL5A2**	NM_000393.3
**HOXA10**	NM_018951.3	**SMO**	NM_005631.3	**FGF5**	NM_004464.3
**JAK3**	NM_000215.2	**TSPAN7**	NM_004615.3	**FUBP1**	NM_003902.3
**KDM6A**	NM_021140.2	**WNT10B**	NM_003394.2	**GNGT1**	NM_021955.3
**MECOM**	NM_005241.2	**CBL**	NM_005188.2	**IDH1**	NM_005896.2
**NTF3**	NM_002527.4	**CREBBP**	NM_004380.2	**RASGRP2**	NM_001098670.1
**TNFRSF10C**	NM_003841.2	**DLL3**	NM_203486.2	**RNF43**	NM_017763.4
**CIC**	NM_015125.3	**EP300**	NM_001429.2	**TTK**	NM_003318.3
**FGF12**	NM_004113.4	**FGFR2**	NM_000141.4	**AXIN1**	NM_181050.1
**HDAC11**	NM_024827.3	**GLI3**	NM_000168.5	**BIRC7**	NM_022161.2
**IFNA7**	NM_021057.2	**LIG4**	NM_002312.3	**CDKN2C**	NM_001262.2
**LIFR**	NM_002310.3	**NUMBL**	NM_004756.3	**CUL1**	NM_003592.2
**NF2**	NM_181828.2	**PHF6**	NM_032335.3	**ERCC6**	NM_000124.2
**PTPN5**	NM_001039970.1	**PIK3CB**	NM_006219.1	**FOXL2**	NM_023067.2
**EIF4EBP1**	NM_004095.3	**PIK3CD**	NM_005026.3	**FZD3**	NM_017412.2

**HSPA6**	NM_002155.3	**SMARCB1**	NM_003073.3	**GADD45B**	NM_015675.2
**IL13RA2**	NM_000640.2	**STAG2**	NM_001042749.1	**ITGB3**	NM_000212.2
**PLA2G3**	NM_015715.3	**TRAF7**	NM_032271.2	**LAMA5**	NM_005560.3
**TGFB3**	NM_003239.2	**ANGPT1**	NM_001146.3	**LEF1**	NM_016269.3
**APC**	NM_000038.3	**CACNB2**	NM_000724.3	**NRAS**	NM_002524.3
**ARID2**	NM_152641.2	**CDK4**	NM_000075.2	**NSD1**	NM_022455.4
**ETS2**	NM_005239.4	**DKK1**	NM_012242.2	**PAX5**	NM_016734.1
**FBXW7**	NM_018315.4	**IRS1**	NM_005544.2	**PPARGC1A**	NM_013261.3
**ID1**	NM_002165.2	**JAG2**	NM_145159.1	**SPP1**	NM_000582.2
**PRKAR2A**	NM_004157.2	**MLLT3**	NM_004529.2	**VEGFA**	NM_001025366.1
**PTTG2**	NM_006607.2	**PAX8**	NM_013953.3	**ALKBH3**	NM_139178.3
**SHC3**	NM_016848.5	**TNF**	NM_000594.2	**COL4A4**	NM_000092.4
**CCNA1**	NM_003914.3	**POLR2D**	NM_004805.3	**HSPA2**	NM_021979.3
**FGF13**	NM_033642.1	**TNFRSF10A**	NM_003844.2	**IL1R1**	NM_000877.2
**GNG7**	NM_052847.1	**BMP6**	NM_001718.2	**MLH1**	NM_000249.2
**IRAK2**	NM_001570.3	**CCNE2**	NM_057735.1	**RASAL1**	NM_004658.1
**ARNT2**	NM_014862.3	**CDKN2A**	NM_000077.3	**RXRG**	NM_006917.3
**FANCF**	NM_022725.2	**DDIT4**	NM_019058.2	**SIN3A**	NM_015477.1
**MCM2**	NM_004526.2	**DTX3**	NM_178502.2	**TLR4**	NM_138554.2
**PRKCA**	NM_002737.2	**FLT1**	NM_002019.4	**WNT5B**	NM_032642.2
**RFC3**	NM_002915.3	**IKBKG**	NM_003639.2	**ARID1A**	NM_006015.4
**SF3B1**	NM_001005526.1	**IL24**	NM_181339.1	**BAMBI**	NM_012342.2
**SMC3**	NM_005445.3	**ITGB6**	NM_001282353.1	**CSF3R**	NM_156038.2
**FGF8**	NM_033163.3	**MFNG**	NM_002405.2	**GLI1**	NM_005269.1
**GSK3B**	NM_002093.2	**SPRY4**	NM_030964.3	**HES5**	NM_001010926.3
**MAP2K6**	NM_002758.3	**ZIC2**	NM_007129.2	**IL7**	NM_000880.2
**PPP2R2C**	NM_181876.2	**POLB**	NM_002690.1	**PRPF38A**	NM_032864.3
**PRKAR1B**	NM_001164759.1	**TSLP**	NM_033035.4	**TRIM39**	NM_021253.3
**SMAD9**	NM_005905.2	**ARID1B**	NM_020732.3	**TLK2**	NM_006852.2
**WNT10A**	NM_025216.2	**BAP1**	NM_004656.2	**EIF2B4**	NM_172195.3
**AKT3**	NM_181690.1	**BMP8A**	NM_181809.3	**C10orf76**	NM_024541.2
**ASXL1**	NM_001164603.1	**CCNB1**	NM_031966.2	**SF3A3**	NM_006802.2
**ATR**	NM_001184.2	**CLCF1**	NM_013246.2	**CC2D1B**	NM_032449.2

**FGF16**	NM_003868.1	**FGF10**	NM_004465.1	**COG7**	NM_153603.3
**FZD10**	NM_007197.2	**FST**	NM_006350.2	**ZNF346**	NM_012279.2
**ITGA8**	NM_003638.1	**HES1**	NM_005524.2	**HDAC3**	NM_003883.2
**MMP3**	NM_002422.3	**HSP90B1**	NM_003299.1	**VPS33B**	NM_018668.3
**NFKB1**	NM_003998.2	**LEFTY2**	NM_003240.2	**SLC4A1AP**	NM_018158.2
**PLCG2**	NM_002661.2	**MAP3K5**	NM_005923.3	**DDX50**	NM_024045.1
**SFRP1**	NM_003012.3	**MLLT4**	NM_005936.2	**CNOT10**	NM_001256741.1
**WNT7A**	NM_004625.3	**MSH2**	NM_000251.1	**MTMR14**	NM_022485.3
**BAD**	NM_004322.3	**PGF**	NM_002632.5	**MRPS5**	NM_031902.3
**BCOR**	NM_001123383.1	**PITX2**	NM_000325.5	**CNOT4**	NM_001190848.1
**CHEK1**	NM_001114121.1	**PTPRR**	NM_001207015.1	**ZC3H14**	NM_001160103.1
**EGFR**	NM_201282.1	**SGK2**	NM_170693.1	**TMUB2**	NM_024107.2
**IRAK3**	NM_007199.1	**TLR2**	NM_003264.3	**DNAJC14**	NM_032364.5
**KRAS**	NM_004985.3	**NOL7**	NM_016167.3	**ZNF143**	NM_003442.5
**MAML2**	NM_032427.1	**FTSJ2**	NM_013393.1	**NUBP1**	NM_001278506.1
**MAP3K14**	NM_003954.1	**TTC31**	NR_027749.1	**PIK3R4**	NM_014602.1
**MCM4**	NM_182746.1	**FCF1**	NM_015962.4		
**PBX1**	NM_002585.2	**PIAS1**	NM_016166.1		

Table S2. Transcripts identified in EV-mRNA from control plasma sample above the background

Gene	RefSEQ	Gene	RefSEQ	Gene	RefSEQ
**RPS27A**	NM_002954.5	**BRAF**	NM_004333.3	**XRCC4**	NM_003401.3
**B2M**	NM_004048.2	**TBL1XR1**	NM_024665.4	**CD40**	NM_001250.4
**H3F3A**	NM_002107.3	**AKT1**	NM_005163.2	**PRKDC**	NM_006904.6
**SKP1**	NM_170679.2	**NBN**	NM_001024688.1	**BID**	NM_197966.1
**RBX1**	NM_014248.2	**SOS1**	NM_005633.2	**MYC**	NM_002467.3
**HIST1H3H**	NM_003536.2	**STAT1**	NM_007315.2	**SMARCA4**	NM_003072.3
**RHOA**	NM_001664.2	**TNFSF10**	NM_003810.2	**TSPAN7**	NM_004615.3
**RAC2**	NM_002872.3	**AKT2**	NM_001626.2	**RELA**	NM_021975.3

**GNAS**	NM_080425.1	**CDKN2D**	NM_001800.3	**PBRM1**	NM_181042.3
**PTEN**	NM_000314.3	**PRKACB**	NM_182948.2	**CDC14A**	NM_033313.2
**ID2**	NM_002166.4	**H3F3C**	NM_001013699.2	**PIK3CA**	NM_006218.2
**PRKAR2B**	NM_002736.2	**MAP2K1**	NM_002755.2	**UBB**	NM_018955.2
**GZMB**	NM_004131.3	**PCNA**	NM_002592.2	**MAPK9**	NM_139068.2
**HHEX**	NM_002729.4	**SOS2**	NM_006939.2	**RFC4**	NM_181573.2
**CDKN1C**	NM_000076.2	**IL1B**	NM_000576.2	**IL24**	NM_181339.1
**TFDP1**	NM_007111.4	**CCND2**	NM_001759.2	**MAPK8**	NM_002750.2
**TET2**	NM_001127208.2	**CDC14B**	NM_003671.3	**CCND1**	NM_053056.2
**GADD45A**	NM_001924.2	**VHL**	NM_000551.2	**PPP3R1**	NM_000945.3
**MYD88**	NM_002468.3	**RAC1**	NM_198829.1	**PTPN11**	NM_002834.3
**JAK1**	NM_002227.1	**IL15**	NM_172174.1	**PPP3CA**	NM_000944.4
**SHC1**	NM_183001.4	**COL2A1**	NM_001844.4	**MAD2L2**	NM_001127325.1
**MAPK1**	NM_138957.2	**PRKCB**	NM_212535.1	**SMC1A**	NM_006306.2
**DUSP6**	NM_001946.2	**SIN3A**	NM_015477.1	**KDM6A**	NM_021140.2
**NFE2L2**	NM_006164.3	**MAP3K1**	NM_005921.1	**PIK3CB**	NM_006219.1
**LEF1**	NM_016269.3	**POLR2H**	NM_001278698.1	**RUNX1T1**	NM_004349.2
**NCOR1**	NM_006311.3	**CAPN2**	NM_001748.4	**CDC7**	NM_003503.2
**AKT3**	NM_181690.1	**CYLD**	NM_015247.1	**PIK3R1**	NM_181504.2
**HDAC2**	NM_001527.1	**PIM1**	NM_002648.2	**UBE2T**	NM_014176.3
**PPP2CB**	NM_001009552.1	**FLNA**	NM_001456.3	**PLA2G4C**	NM_003706.2
**CCND3**	NM_001760.2	**GSK3B**	NM_002093.2	**RASGRP1**	NM_005739.3
**KAT2B**	NM_003884.3	**KLF4**	NM_004235.4	**CDC25B**	NM_021873.2
**SETD2**	NM_014159.6	**SMAD4**	NM_005359.3	**ALKBH3**	NM_139178.3
**RRAS2**	NM_001102669.2	**CDKN1A**	NM_000389.2	**PRKACA**	NM_002730.3
**TCF3**	NM_003200.2	**RB1**	NM_000321.1	**SMAD2**	NM_001003652.1
**RAD21**	NM_006265.2	**SMC3**	NM_005445.3	**MAP2K4**	NM_003010.2
**BCL2L1**	NM_138578.1	**HDAC1**	NM_004964.2	**PBX3**	NM_006195.5
**TGFB1**	NM_000660.3	**CASP3**	NM_032991.2	**NRAS**	NM_002524.3
**BCL2A1**	NM_004049.2	**LTBP1**	NM_000627.3	**CD19**	NM_001770.4
**U2AF1**	NM_001025203.1	**PPP2R1A**	NM_014225.3	**CEBPE**	NM_001805.2
**WHSC1L1**	NM_017778.2	**RUNX1**	NM_001754.4	**GRB2**	NM_002086.4
**GNAQ**	NM_002072.2	**ITGB3**	NM_000212.2	**PHF6**	NM_032335.3

**NFKBIA**	NM_020529.1	**MDM2**	NM_006878.2	**STAT4**	NM_003151.2
**PIK3CG**	NM_002649.2	**IGF1**	NM_000618.3	**CREB5**	NM_182898.2
**MYB**	NM_005375.2	**TP53**	NM_000546.2	**MAP2K6**	NM_002758.3
**CCNA2**	NM_001237.2	**CCNB1**	NM_031966.2	**MECOM**	NM_005241.2
**APC**	NM_000038.3	**FGF2**	NM_002006.4	**MLH1**	NM_000249.2
**FOS**	NM_005252.2	**BRIP1**	NM_032043.1	**PML**	NM_002675.3
**SOCS2**	NM_003877.3	**GTF2H3**	NM_001516.3	**IDH1**	NM_005896.2
**IFNG**	NM_000619.2	**KMT2C**	NM_170606.2	**PLCG2**	NM_002661.2
**CHUK**	NM_001278.3	**ARNT2**	NM_014862.3	**SPOP**	NM_001007226.1
**MLLT3**	NM_004529.2	**FLT1**	NM_002019.4	**MDC1**	NM_014641.2
**STK11**	NM_000455.4	**FUBP1**	NM_003902.3	**PRKCA**	NM_002737.2
**FOXO4**	NM_005938.2	**C19orf40**	NM_152266.3	**BAD**	NM_004322.3
**THBS1**	NM_003246.2	**DDIT3**	NM_004083.4	**FGF12**	NM_004113.4
**TNFAIP3**	NM_006290.2	**EZH2**	NM_004456.3	**HSPB1**	NM_001540.3
**BMP6**	NM_001718.2	**PDGFA**	NM_002607.5	**PPP3CC**	NM_005605.3
**HSP90B1**	NM_003299.1	**SMO**	NM_005631.3	**TLR4**	NM_138554.2
**IL13RA2**	NM_000640.2	**DLL3**	NM_203486.2	**TRAF7**	NM_032271.2
**CBL**	NM_005188.2	**FANCL**	NM_001114636.1	**TIAM1**	NM_003253.2
**BNIP3**	NM_004052.2	**BAX**	NM_138761.3	**ALKBH2**	NM_001001655.2
**POLB**	NM_002690.1	**PPP3CB**	NM_001142354.1	**EP300**	NM_001429.2
**CTNNB1**	NM_001904.3	**DNMT3A**	NM_022552.3	**MAP3K5**	NM_005923.3
**LEPR**	NM_001003679.1	**DTX4**	NM_015177.1	**RET**	NM_020630.4
**POLD4**	NM_021173.2	**IRS1**	NM_005544.2	**ETS2**	NM_005239.4
**SMAD3**	NM_005902.3	**CSF3R**	NM_156038.2	**IGFBP3**	NM_000598.4
**NFKB1**	NM_003998.2	**HSPA1A**	NM_005345.5	**MCM4**	NM_182746.1
**ARID2**	NM_152641.2	**THEM4**	NM_053055.4	**MFNG**	NM_002405.2
**CASP8**	NM_001228.4	**PRKAR2A**	NM_004157.2	**UTY**	NM_007125.3
**MAPK3**	NM_001040056.1	**CUL1**	NM_003592.2	**BRCA1**	NM_007305.2
**ATRX**	NM_000489.3	**KRAS**	NM_004985.3	**HOXA10**	NM_018951.3
**FAS**	NM_152876.1	**STAT3**	NM_139276.2	**HOXA9**	NM_152739.3
**GNG12**	NM_018841.3	**CREB3L4**	NM_130898.2	**MPL**	NM_005373.2
**POLR2D**	NM_004805.3	**HDAC5**	NM_005474.4	**PPARG**	NM_015869.3
**RAF1**	NM_002880.2	**IRAK2**	NM_001570.3	**TNN**	NM_022093.1

**STAG2**	NM_001042749.1	**LAT**	NM_001014987.1	**NOL7**	NM_016167.3
**XPA**	NM_000380.3	**MEN1**	NM_130802.2	**FTSJ2**	NM_013393.1
**CCNE1**	NM_001238.1	**WT1**	NM_000378.3	**PIAS1**	NM_016166.1
**CEBPA**	NM_004364.2	**CHEK1**	NM_001114121.1	**TTC31**	NR_027749.1
**RPS6KA5**	NM_004755.2	**FEN1**	NM_004111.4	**PRPF38A**	NM_032864.3
**PIK3R5**	NM_001142633.1	**LIG4**	NM_002312.3	**EIF2B4**	NM_172195.3
**JAK2**	NM_004972.2	**MGMT**	NM_002412.3	**MRPS5**	NM_031902.3
**BCL2**	NM_000657.2	**MNAT1**	NM_002431.2	**CNOT4**	NM_001190848.1
**MSH6**	NM_000179.1	**NF1**	NM_000267.2	**CNOT10**	NM_001256741.1
**DUSP5**	NM_004419.3	**SMARCB1**	NM_003073.3	**ZC3H14**	NM_001160103.1
**IL8**	NM_000584.2	**TLR2**	NM_003264.3	**DDX50**	NM_024045.1
**SAP130**	NM_024545.3	**TMUB2**	NM_024107.2	**AGK**	NM_018238.3
**SF3A3**	NM_006802.2	**HDAC3**	NM_003883.2	**USP39**	NM_001256725.1
**MTMR14**	NM_022485.3	**FCF1**	NM_015962.4	**ZNF346**	NM_012279.2
**TRIM39**	NM_021253.3	**C10orf76**	NM_024541.2	**COG7**	NM_153603.3
**ZNF143**	NM_003442.5	**TLK2**	NM_006852.2	**RBM45**	NM_152945.2
				**ZNF384**	NM_133476.3

Table S3. Transcripts identified in EV-mRNA from BPH plasma sample above the background

Gene	RefSEQ	Gene	RefSEQ	Gene	RefSEQ
**RPS27A**	NM_002954.5	**RRAS2**	NM_001102669.2	**CYLD**	NM_015247.1
**H3F3A**	NM_002107.3	**H3F3C**	NM_001013699.2	**MSH6**	NM_000179.1
**SKP1**	NM_170679.2	**LEF1**	NM_016269.3	**GSK3B**	NM_002093.2
**B2M**	NM_004048.2	**IL1B**	NM_000576.2	**BRAF**	NM_004333.3
**RBX1**	NM_014248.2	**TBL1XR1**	NM_024665.4	**CCND2**	NM_001759.2
**PTEN**	NM_000314.3	**NFKBIA**	NM_020529.1	**CDC7**	NM_003503.2
**RHOA**	NM_001664.2	**KAT2B**	NM_003884.3	**CHUK**	NM_001278.3
**CDKN1C**	NM_000076.2	**PLA2G4C**	NM_003706.2	**TSPAN7**	NM_004615.3
**HHEX**	NM_002729.4	**PPP2CB**	NM_001009552.1	**VHL**	NM_000551.2
**ID2**	NM_002166.4	**BCL2A1**	NM_004049.2	**MAP2K4**	NM_003010.2
**TET2**	NM_001127208.2	**KLF4**	NM_004235.4	**MAD2L2**	NM_001127325.1
**RAC2**	NM_002872.3	**WHSC1L1**	NM_017778.2	**MYB**	NM_005375.2
**GADD45A**	NM_001924.2	**JAK1**	NM_002227.1	**PHF6**	NM_032335.3

**DUSP6**	NM_001946.2	**AKT1**	NM_005163.2	**AKT3**	NM_181690.1
**TFDP1**	NM_007111.4	**RAD21**	NM_006265.2	**RUNX1**	NM_001754.4
**HIST1H3H**	NM_003536.2	**HDAC1**	NM_004964.2	**TNFSF10**	NM_003810.2
**SHC1**	NM_183001.4	**MAP2K1**	NM_002755.2	**BID**	NM_197966.1
**TCF3**	NM_003200.2	**COL2A1**	NM_001844.4	**MLH1**	NM_000249.2
**NFE2L2**	NM_006164.3	**SMARCA4**	NM_003072.3	**PIM1**	NM_002648.2
**GNAS**	NM_080425.1	**NCOR1**	NM_006311.3	**PTCH1**	NM_000264.3
**GZMB**	NM_004131.3	**UBB**	NM_018955.2	**CDKN1A**	NM_000389.2
**HDAC2**	NM_001527.1	**NBN**	NM_001024688.1	**CASP8**	NM_001228.4
**PRKAR2B**	NM_002736.2	**BCL2L1**	NM_138578.1	**CD40**	NM_001250.4
**STAT1**	NM_007315.2	**CCND3**	NM_001760.2	**RFC4**	NM_181573.2
**MYD88**	NM_002468.3	**CEBPE**	NM_001805.2	**DUSP5**	NM_004419.3
**U2AF1**	NM_001025203.1	**AKT2**	NM_001626.2	**FLNA**	NM_001456.3
**GNAQ**	NM_002072.2	**MYC**	NM_002467.3	**TNFAIP3**	NM_006290.2
**IL15**	NM_172174.1	**IL13RA2**	NM_000640.2	**TIAM1**	NM_003253.2
**TGFB1**	NM_000660.3	**RB1**	NM_000321.1	**MDM2**	NM_006878.2
**CCND1**	NM_053056.2	**CASP3**	NM_032991.2	**E2F1**	NM_005225.1
**SETD2**	NM_014159.6	**CD19**	NM_001770.4	**FOS**	NM_005252.2
**RASGRP1**	NM_005739.3	**MTOR**	NM_004958.2	**KDM6A**	NM_021140.2
**SMAD3**	NM_005902.3	**PBRM1**	NM_181042.3	**PIK3R5**	NM_001142633.1
**PRKDC**	NM_006904.6	**CEBPA**	NM_004364.2	**JAG2**	NM_145159.1
**CAPN2**	NM_001748.4	**DNMT3A**	NM_022552.3	**NTF3**	NM_002527.4
**PDGFA**	NM_002607.5	**LEP**	NM_000230.2	**AXIN1**	NM_181050.1
**SOS2**	NM_006939.2	**NR4A1**	NM_173157.1	**JAK2**	NM_004972.2
**PPP2R1A**	NM_014225.3	**BAX**	NM_138761.3	**PAX5**	NM_016734.1
**RUNX1T1**	NM_004349.2	**FGF2**	NM_002006.4	**PIK3CG**	NM_002649.2
**SIN3A**	NM_015477.1	**MCM5**	NM_006739.3	**PIK3R1**	NM_181504.2
**MAP3K1**	NM_005921.1	**DAXX**	NM_001350.3	**POLB**	NM_002690.1
**SMC3**	NM_005445.3	**EIF4EBP1**	NM_004095.3	**POLR2D**	NM_004805.3
**SOS1**	NM_005633.2	**SMAD2**	NM_001003652.1	**PPP3R1**	NM_000945.3
**GTF2H3**	NM_001516.3	**BRIP1**	NM_032043.1	**PTPRR**	NM_001207015.1
**STK11**	NM_000455.4	**CDKN2D**	NM_001800.3	**SIRT4**	NM_012240.1
**POLR2H**	NM_001278698.1	**IRAK2**	NM_001570.3	**SOCS2**	NM_003877.3

**RPS6KA5**	NM_004755.2	**MAPK1**	NM_138957.2	**TGFBR2**	NM_001024847.1
**XRCC4**	NM_003401.3	**PCNA**	NM_002592.2	**UBE2T**	NM_014176.3
**ATR**	NM_001184.2	**PRKCB**	NM_212535.1	**ZAK**	NM_016653.2
**GRB2**	NM_002086.4	**STAT4**	NM_003151.2	**FTSJ2**	NM_013393.1
**IL24**	NM_181339.1	**ALKBH3**	NM_139178.3	**NOL7**	NM_016167.3
**PRKAR2A**	NM_004157.2	**HDAC11**	NM_024827.3	**PIAS1**	NM_016166.1
**TRAF7**	NM_032271.2	**HOXA10**	NM_018951.3	**TTC31**	NR_027749.1
**TTK**	NM_003318.3	**HPGD**	NM_001145816.2	**MRPS5**	NM_031902.3
**CBL**	NM_005188.2	**MAPK3**	NM_001040056.1	**TRIM39**	NM_021253.3
**CCNE1**	NM_001238.1	**PIK3CB**	NM_006219.1	**FCF1**	NM_015962.4
**CREB5**	NM_182898.2	**PRKACA**	NM_002730.3	**EIF2B4**	NM_172195.3
**FGF23**	NM_020638.2	**PRKCA**	NM_002737.2	**PRPF38A**	NM_032864.3
**SMC1A**	NM_006306.2	**RAF1**	NM_002880.2	**CNOT10**	NM_001256741.1
**SMO**	NM_005631.3	**SF3B1**	NM_001005526.1	**COG7**	NM_153603.3
**CREBBP**	NM_004380.2	**SMARCB1**	NM_003073.3	**TMUB2**	NM_024107.2
**PBX3**	NM_006195.5	**ALKBH2**	NM_001001655.2	**ZC3H14**	NM_001160103.1
**PRKACB**	NM_182948.2	**ANGPT1**	NM_001146.3	**CNOT4**	NM_001190848.1
**BNIP3**	NM_004052.2	**DTX4**	NM_015177.1	**ZNF346**	NM_012279.2
**CDC14B**	NM_003671.3	**FGF12**	NM_004113.4	**DDX50**	NM_024045.1
**LTBP1**	NM_000627.3	**ITGB6**	NM_001282353.1	**NUBP1**	NM_001278506.1

Table S4. Transcripts identified in EV-mRNA from mPC urine sample above the background.

Gene	RefSEQ	Gene	RefSEQ	Gene	RefSEQ
**RPS27A**	NM_002954.5	**PGF**	NM_002632.5	**PPP3R1**	NM_000945.3
**IL8**	NM_000584.2	**ENDOG**	NM_004435.2	**TNN**	NM_022093.1
**H3F3A**	NM_002107.3	**TET2**	NM_001127208.2	**PLA2G3**	NM_015715.3
**DUSP8**	NM_004420.2	**DAXX**	NM_001350.3	**TLX1**	NM_005521.3
**IFNA17**	NM_021268.2	**SHC1**	NM_183001.4	**WNT10B**	NM_003394.2
**PTEN**	NM_000314.3	**FANCC**	NM_000136.2	**WNT6**	NM_006522.3
**B2M**	NM_004048.2	**DTX1**	NM_004416.2	**NKD1**	NM_033119.3
**SKP1**	NM_170679.2	**SPRY2**	NM_005842.2	**PRKAR2B**	NM_002736.2
**IL1B**	NM_000576.2	**FGFR3**	NM_022965.2	**AMER1**	NM_152424.3

**GNAQ**	NM_002072.2	**PITX2**	NM_000325.5	**IL11**	NM_000641.2
**H3F3C**	NM_001013699.2	**HMGA2**	NM_003484.1	**PPP3CB**	NM_001142354.1
**BCL2A1**	NM_004049.2	**HDAC11**	NM_024827.3	**TCF3**	NM_003200.2
**FOS**	NM_005252.2	**PDGFB**	NM_033016.2	**MSH6**	NM_000179.1
**PTPN11**	NM_002834.3	**NODAL**	NM_018055.3	**PIK3CG**	NM_002649.2
**GADD45A**	NM_001924.2	**PIM1**	NM_002648.2	**ATR**	NM_001184.2
**SOCS3**	NM_003955.3	**ALKBH2**	NM_001001655.2	**FLT1**	NM_002019.4
**CYLD**	NM_015247.1	**PTCH1**	NM_000264.3	**SOS2**	NM_006939.2
**FLNC**	NM_001127487.1	**BRAF**	NM_004333.3	**WNT5A**	NM_003392.3
**TGFB1**	NM_000660.3	**CDKN2B**	NM_004936.3	**CCND3**	NM_001760.2
**RELA**	NM_021975.3	**FGFR4**	NM_002011.3	**CDC14B**	NM_003671.3
**COL5A1**	NM_000093.3	**HDAC4**	NM_006037.3	**CREB5**	NM_182898.2
**PML**	NM_002675.3	**CD19**	NM_001770.4	**HNF1A**	NM_000545.4
**TCF7L1**	NM_031283.1	**NR4A1**	NM_173157.1	**GNG12**	NM_018841.3
**FGFR1**	NM_015850.2	**RRAS2**	NM_001102669.2	**CDC7**	NM_003503.2
**C19orf40**	NM_152266.3	**FGF23**	NM_020638.2	**DKK4**	NM_014420.2
**GNA11**	NM_002067.1	**CDKN1A**	NM_000389.2	**BRIP1**	NM_032043.1
**TPO**	NM_175722.1	**COL2A1**	NM_001844.4	**SMAD4**	NM_005359.3
**APH1B**	NM_001145646.1	**LEFTY2**	NM_003240.2	**SOS1**	NM_005633.2
**PRKCA**	NM_002737.2	**PPARGC1A**	NM_013261.3	**PLA2G5**	NM_000929.2
**SIRT4**	NM_012240.1	**SOCS1**	NM_003745.1	**VHL**	NM_000551.2
**NFE2L2**	NM_006164.3	**ACVR1B**	NM_004302.3	**HPGD**	NM_001145816.2
**DNMT3A**	NM_022552.3	**POLD4**	NM_021173.2	**JAG2**	NM_145159.1
**NTRK2**	NM_001007097.1	**PRKDC**	NM_006904.6	**SMARCA4**	NM_003072.3
**PIK3R5**	NM_001142633.1	**PBRM1**	NM_181042.3	**PTPRR**	NM_001207015.1
**MUTYH**	NM_012222.2	**WT1**	NM_000378.3	**FGF11**	NM_004112.2
**HDAC5**	NM_005474.4	**PLA2G4C**	NM_003706.2	**SMAD3**	NM_005902.3
**PDGFA**	NM_002607.5	**HOXA9**	NM_152739.3	**DUSP6**	NM_001946.2
**STK11**	NM_000455.4	**NR4A3**	NM_173198.1	**MYD88**	NM_002468.3
**GZMB**	NM_004131.3	**EPOR**	NM_000121.2	**HMGA1**	NM_145904.1
**ITGB6**	NM_001282353.1	**MAP2K4**	NM_003010.2	**SPP1**	NM_000582.2
**STAT1**	NM_007315.2	**IFNA2**	NM_000605.3	**FGF10**	NM_004465.1
**CD40**	NM_001250.4	**SPOP**	NM_001007226.1	**WNT3**	NM_030753.3

**CEBPA**	NM_004364.2	**CREB3L4**	NM_130898.2	**SFRP1**	NM_003012.3
**CDC25B**	NM_021873.2	**FGF12**	NM_004113.4	**CIC**	NM_015125.3
**IRS1**	NM_005544.2	**SP1**	NM_003109.1	**EFNA1**	NM_004428.2
**ITGA9**	NM_002207.2	**SIX1**	NM_005982.3	**NFKBIA**	NM_020529.1
**ARNT2**	NM_014862.3	**IL15**	NM_172174.1	**RASAL1**	NM_004658.1
**IL11RA**	NM_147162.1	**MNAT1**	NM_002431.2	**EP300**	NM_001429.2
**KMT2C**	NM_170606.2	**RBX1**	NM_014248.2	**DTX3**	NM_178502.2
**NSD1**	NM_022455.4	**HSPA2**	NM_021979.3	**KITLG**	NM_003994.4
**DUSP4**	NM_057158.2	**BAMBI**	NM_012342.2	**LIFR**	NM_002310.3
**FZD3**	NM_017412.2	**CD14**	NM_000591.2	**GATA3**	NM_001002295.1
**IGFBP3**	NM_000598.4	**HHEX**	NM_002729.4	**SHC3**	NM_016848.5
**RUNX1T1**	NM_004349.2	**FGFR2**	NM_000141.4	**EPO**	NM_000799.2
**CREBBP**	NM_004380.2	**MAP3K8**	NM_005204.2	**PAK7**	NM_177990.1
**NTF3**	NM_002527.4	**ANGPT1**	NM_001146.3	**EGFR**	NM_201282.1
**EFNA2**	NM_001405.3	**IKBKG**	NM_003639.2	**FGF8**	NM_033163.3
**FZD8**	NM_031866.1	**SPRY1**	NM_005841.1	**ERCC6**	NM_000124.2
**IL7R**	NM_002185.2	**FST**	NM_006350.2	**SETD2**	NM_014159.6
**SMAD2**	NM_001003652.1	**POLR2D**	NM_004805.3	**CDKN2D**	NM_001800.3
**MCM2**	NM_004526.2	**TNFRSF10C**	NM_003841.2	**CLCF1**	NM_013246.2
**BCL2**	NM_000657.2	**HOXA11**	NM_005523.5	**PBX3**	NM_006195.5
**COL5A2**	NM_000393.3	**NUMBL**	NM_004756.3	**WNT10A**	NM_025216.2
**CASP8**	NM_001228.4	**TIAM1**	NM_003253.2	**CACNB2**	NM_000724.3
**SMAD9**	NM_005905.2	**DTX4**	NM_015177.1	**PLA2G4F**	NM_213600.2
**IFNG**	NM_000619.2	**MYB**	NM_005375.2	**CEBPE**	NM_001805.2
**IL1A**	NM_000575.3	**MPL**	NM_005373.2	**FGF13**	NM_033642.1
**BMP2**	NM_001200.2	**RAD50**	NM_005732.2	**GHR**	NM_000163.2
**MET**	NM_000245.2	**MMP3**	NM_002422.3	**COL4A4**	NM_000092.4
**PRKAA2**	NM_006252.2	**APC**	NM_000038.3	**LIG4**	NM_002312.3
**CALML3**	NM_005185.2	**CNTFR**	NM_147164.1	**RAC2**	NM_002872.3
**CHEK2**	NM_007194.3	**MYC**	NM_002467.3	**PIK3CB**	NM_006219.1
**FEN1**	NM_004111.4	**BMP6**	NM_001718.2	**GAS1**	NM_002048.2
**MAPK1**	NM_138957.2	**COL1A1**	NM_000088.3	**BIRC7**	NM_022161.2
**DLL3**	NM_203486.2	**PDGFC**	NM_016205.1	**NPM1**	NM_002520.5

**BDNF**	NM_170732.4	**COL11A1**	NM_001854.3	**THEM4**	NM_053055.4
**CHUK**	NM_001278.3	**IL13**	NM_002188.2	**MAP2K1**	NM_002755.2
**POLR2J**	NM_006234.4	**TGFB3**	NM_003239.2	**MEN1**	NM_130802.2
**BCL2L1**	NM_138578.1	**CBL**	NM_005188.2	**ARID1A**	NM_006015.4
**LAT**	NM_001014987.1	**LEPR**	NM_001003679.1	**MECOM**	NM_005241.2
**SMARCB1**	NM_003073.3	**MAP3K13**	NM_004721.3	**PCNA**	NM_002592.2
**BRCA1**	NM_007305.2	**ACVR2A**	NM_001616.3	**ITGA8**	NM_003638.1
**FGF14**	NM_004115.3	**PTPN5**	NM_001039970.1	**PAX5**	NM_016734.1
**COL4A3**	NM_000091.3	**ZIC2**	NM_007129.2	**BMP5**	NM_021073.2
**WHSC1**	NM_007331.1	**NRAS**	NM_002524.3	**FGF5**	NM_004464.3
**SOCS2**	NM_003877.3	**PAX8**	NM_013953.3	**GLI3**	NM_000168.5
**TNF**	NM_000594.2	**PIK3CA**	NM_006218.2	**BID**	NM_197966.1
**GNG7**	NM_052847.1	**PRKACG**	NM_002732.2	**NGFR**	NM_002507.1
**MTOR**	NM_004958.2	**CCR7**	NM_001838.2	**CCND1**	NM_053056.2
**DUSP5**	NM_004419.3	**PPP2R2C**	NM_181876.2	**ID1**	NM_002165.2
**FANCB**	NM_152633.2	**RXRG**	NM_006917.3	**BNIP3**	NM_004052.2
**PPARG**	NM_015869.3	**FBXW7**	NM_018315.4	**CDKN2A**	NM_000077.3
**ACVR1C**	NM_145259.2	**IL1R1**	NM_000877.2	**GLI1**	NM_005269.1
**PIK3R3**	NM_003629.3	**MAPK10**	NM_002753.2	**CACNG6**	NM_145814.1
**SRSF2**	NM_003016.3	**HSPB1**	NM_001540.3	**ID4**	NM_001546.2
**FOXO4**	NM_005938.2	**IFNA7**	NM_021057.2	**MAP2K6**	NM_002758.3
**TNFSF10**	NM_003810.2	**MDC1**	NM_014641.2	**IL20RB**	NM_144717.2
**IL7**	NM_000880.2	**HSPA6**	NM_002155.3	**KRAS**	NM_004985.3
**TSLP**	NM_033035.4	**MAML2**	NM_032427.1	**U2AF1**	NM_001025203.1
**TFDP1**	NM_007111.4	**RPS6KA6**	NM_014496.1	**IDH2**	NM_002168.2
**DKK1**	NM_012242.2	**ITGA7**	NM_002206.1	**HDAC2**	NM_001527.1
**SOX9**	NM_000346.2	**AXIN1**	NM_181050.1	**DDIT3**	NM_004083.4
**PRKAR1B**	NM_001164759.1	**SFN**	NM_006142.3	**ITGB3**	NM_000212.2
**SOX17**	NM_022454.3	**SPRY4**	NM_030964.3	**RNF43**	NM_017763.4
**WNT5B**	NM_032642.2	**BCOR**	NM_001123383.1	**HES5**	NM_001010926.3
**LEP**	NM_000230.2	**FZD9**	NM_003508.2	**GATA2**	NM_032638.3
**PRMT8**	NM_019854.3	**MLF1**	NM_022443.3	**IL2RB**	NM_000878.2
**COL4A5**	NM_033381.1	**IL5RA**	NM_000564.3	**RAD21**	NM_006265.2

**FGF9**	NM_002010.2	**MLLT3**	NM_004529.2	**OSM**	NM_020530.3
**IL24**	NM_181339.1	**STAT3**	NM_139276.2	**UBB**	NM_018955.2
**RB1**	NM_000321.1	**EFNA5**	NM_001962.2	**TNFAIP3**	NM_006290.2
**NF2**	NM_181828.2	**HSPA1A**	NM_005345.5	**MCM7**	NM_182776.1
**HIST1H3G**	NM_003534.2	**CACNA1E**	NM_000721.2	**TTK**	NM_003318.3
**MLLT4**	NM_005936.2	**NBN**	NM_001024688.1	**EGF**	NM_001963.3
**RHOA**	NM_001664.2	**FOXL2**	NM_023067.2	**FANCF**	NM_022725.2
**DUSP10**	NM_144728.2	**CDKN1C**	NM_000076.2	**SFRP2**	NM_003013.2
**TNFRSF10A**	NM_003844.2	**KDM6A**	NM_021140.2	**LIF**	NM_002309.3
**PPP3CA**	NM_000944.4	**PPP2CB**	NM_001009552.1	**ARID1B**	NM_020732.3
**IGF1**	NM_000618.3	**ETS2**	NM_005239.4	**BMP8A**	NM_181809.3
**PRKAR2A**	NM_004157.2	**GNGT1**	NM_021955.3	**WHSC1L1**	NM_017778.2
**FAS**	NM_152876.1	**PAK3**	NM_002578.2	**PROM1**	NM_006017.1
**POLR2H**	NM_001278698.1	**ABL1**	NM_005157.3	**PLAT**	NM_000931.2
**WNT16**	NM_057168.1	**HIST1H3H**	NM_003536.2	**CACNB4**	NM_001005747.2
**KLF4**	NM_004235.4	**LAMA5**	NM_005560.3	**CASP10**	NM_032977.3
**PIK3R1**	NM_181504.2	**FGF16**	NM_003868.1	**FZD10**	NM_007197.2
**FGF21**	NM_019113.2	**GRIN2A**	NM_000833.3	**IBSP**	NM_004967.3
**RAC1**	NM_198829.1	**ETV4**	NM_001079675.1	**IGF1R**	NM_000875.2
**CAPN2**	NM_001748.4	**IL13RA2**	NM_000640.2	**CDC14A**	NM_033313.2
**TLR4**	NM_138554.2	**RUNX1**	NM_001754.4	**CSF3R**	NM_156038.2
**DUSP2**	NM_004418.3	**HES1**	NM_005524.2	**CSF3**	NM_000759.3
**LAMA3**	NM_000227.3	**IL12RB2**	NM_001559.2	**LAMC3**	NM_006059.3
**PPP3R2**	NM_147180.2	**SF3B1**	NM_001005526.1	**MFNG**	NM_002405.2
**VEGFA**	NM_001025366.1	**MMP7**	NM_002423.3	**MLH1**	NM_000249.2
**COL6A6**	NM_001102608.1	**MAP3K14**	NM_003954.1	**PRKACB**	NM_182948.2
**CXXC4**	NM_025212.1	**NCOR1**	NM_006311.3	**MAPK8**	NM_002750.2
**IL12B**	NM_002187.2	**CDKN1B**	NM_004064.2	**FCF1**	NM_015962.4
**IL1R2**	NM_173343.1	**CTNNB1**	NM_001904.3	**PRPF38A**	NM_032864.3
**CCNE1**	NM_001238.1	**RAF1**	NM_002880.2	**TLK2**	NM_006852.2
**SMC3**	NM_005445.3	**CALML5**	NM_017422.4	**CC2D1B**	NM_032449.2
**GPC4**	NM_001448.2	**KAT2B**	NM_003884.3	**SF3A3**	NM_006802.2
**PDGFRB**	NM_002609.3	**H2AFX**	NM_002105.2	**ZNF346**	NM_012279.2

**CDK4**	NM_000075.2	**MYCN**	NM_005378.4	**TRIM39**	NM_021253.3
**ETV1**	NM_004956.4	**WNT7B**	NM_058238.1	**TTC31**	NR_027749.1
**FZD7**	NM_003507.1	**SIN3A**	NM_015477.1	**FTSJ2**	NM_013393.1
**NASP**	NM_172164.1	**AMH**	NM_000479.3	**CNOT10**	NM_001256741.1
**AKT1**	NM_005163.2	**FGF2**	NM_002006.4	**C10orf76**	NM_024541.2
**FASLG**	NM_000639.1	**IL22RA1**	NM_021258.2	**VPS33B**	NM_018668.3
**PAX3**	NM_013942.3	**SGK2**	NM_170693.1	**COG7**	NM_153603.3
**GADD45B**	NM_015675.2	**UTY**	NM_007125.3	**DDX50**	NM_024045.1
**JUN**	NM_002228.3	**GRB2**	NM_002086.4	**SLC4A1AP**	NM_018158.2
**HOXA10**	NM_018951.3	**MGMT**	NM_002412.3	**ZC3H14**	NM_001160103.1
**ARID2**	NM_152641.2	**CASP9**	NM_001229.2	**PIAS1**	NM_016166.1
**RFC4**	NM_181573.2	**HIST1H3B**	NM_003537.3	**HDAC3**	NM_003883.2
**CCND2**	NM_001759.2	**PLAU**	NM_002658.2	**TMUB2**	NM_024107.2
**TNR**	NM_003285.2	**TRAF7**	NM_032271.2	**DNAJC14**	NM_032364.5
**GRIA3**	NM_000828.4	**ATRX**	NM_000489.3	**AMECR1L**	NM_001199140.1
**WNT2**	NM_003391.2	**CARD11**	NM_032415.2	**NOL7**	NM_016167.3
**PCK1**	NM_002591.2	**CDC6**	NM_001254.3	**CNOT4**	NM_001190848.1
**RPA3**	NM_002947.3	**TGFBR2**	NM_001024847.1	**MRPS5**	NM_031902.3
**GNAS**	NM_080425.1	**PPP2R1A**	NM_014225.3	**GPATCH3**	NM_022078.2
**CACNG4**	NM_014405.2	**IL2RA**	NM_000417.1	**ZKSCAN5**	NM_014569.3
**GATA1**	NM_002049.2	**BAX**	NM_138761.3	**EIF2B4**	NM_172195.3
**XRCC4**	NM_003401.3	**CSF2**	NM_000758.2	**ZNF384**	NM_133476.3
